# East Eurasian ancestry in the middle of Europe: genetic footprints of Steppe nomads in the genomes of Belarusian Lipka Tatars

**DOI:** 10.1038/srep30197

**Published:** 2016-07-25

**Authors:** Vasili Pankratov, Sergei Litvinov, Alexei Kassian, Dzmitry Shulhin, Lieve Tchebotarev, Bayazit Yunusbayev, Märt Möls, Hovhannes Sahakyan, Levon Yepiskoposyan, Siiri Rootsi, Ene Metspalu, Maria Golubenko, Natalia Ekomasova, Farida Akhatova, Elza Khusnutdinova, Evelyne Heyer, Phillip Endicott, Miroslava Derenko, Boris Malyarchuk, Mait Metspalu, Oleg Davydenko, Richard Villems, Alena Kushniarevich

**Affiliations:** 1 Institute of Genetics and Cytology, National Academy of Sciences of Belarus, Minsk, Belarus; 2 Institute of Biochemistry and Genetics, Ufa Research Centre, RAS, Ufa, Bashkortostan, Russia; 3 Estonian Biocentre, Tartu, Estonia; 4 Institute of Linguistics, Russian Academy of Sciences, Moscow, Russia; 5 School for Advanced Studies in the Humanities, Russian Presidential Academy of National Economy and Public Administration, Moscow, Russia; 6 Belarusian State University, Faculty of Applied Mathematics and Computer Science Department of Probability Theory and Mathematical Statistics, Minsk, Belarus; 7 Center of analytical and genetic engineering studies, Institute of Microbiology, National Academy of Sciences of Belarus, Minsk, Belarus; 8 Institute of Mathematical Statistics, University of Tartu, Tartu, Estonia; 9 Laboratory of Ethnogenomics, Institute of Molecular Biology, National Academy of Sciences of Armenia, Yerevan, 0014, Armenia; 10 Department of Evolutionary Biology, Institute of Molecular and Cell Biology, University of Tartu, Tartu, Estonia; 11 The Research Institute for Medical Genetics, 634050, Tomsk, Russia; 12 Department of Genetics and Fundamental Medicine of Bashkir State University, Ufa, Bashkortostan, Russia; 13 Institute of Fundamental Medicine and Biology, Kazan Federal University, Kazan, Russia; 14 Eco-Anthropologie et Ethnobiologie, UMR 7206 CNRS, MNHN, Université Paris Diderot, Sorbonne Universités, Muséum national d’Histoire naturelle, Musée de l’Homme, Paris, France; 15 Institute of Biological Problems of the North, Russian Academy of Sciences, Magadan, Russia

## Abstract

Medieval era encounters of nomadic groups of the Eurasian Steppe and largely sedentary East Europeans had a variety of demographic and cultural consequences. Amongst these outcomes was the emergence of the Lipka Tatars—a Slavic-speaking Sunni-Muslim minority residing in modern Belarus, Lithuania and Poland, whose ancestors arrived in these territories via several migration waves, mainly from the Golden Horde. Our results show that Belarusian Lipka Tatars share a substantial part of their gene pool with Europeans as indicated by their Y-chromosomal, mitochondrial and autosomal DNA variation. Nevertheless, Belarusian Lipkas still retain a strong genetic signal of their nomadic ancestry, witnessed by the presence of common Y-chromosomal and mitochondrial DNA variants as well as autosomal segments identical by descent between Lipkas and East Eurasians from temperate and northern regions. Hence, we document Lipka Tatars as a unique example of former Medieval migrants into Central Europe, who became sedentary, changed language to Slavic, yet preserved their faith and retained, both uni- and bi-parentally, a clear genetic echo of a complex population interplay throughout the Eurasian Steppe Belt, extending from Central Europe to northern China.

Medieval migrations of Turkic-speaking nomads constitute a series of massive migration events in the history of Eurasia. They led to the spread of Turkic languages over a vast area, ranging from East Europe and Anatolia in the West to East and North Siberia in the East^1^. These migrations, besides their cultural influence, left a detectable impact on the genetic landscape of Eurasia: almost all extant Turkic peoples carry in their genomes DNA segments, though in different amounts, which they share with populations from South Siberia and Mongolia^2^. Despite this common feature, the genetic and demographic histories of Turkic populations can differ considerably. As far as East Europe is concerned, many of the mighty, largely Turkic-speaking tribes and confederations thereof, – such as Khazars, Volga Bulgars and Cumans, who once dominated the Ponto-Caspian steppes and beyond — already vanished from political and ethnic maps long ago; whereas such as Volga Tatars, Chuvashes, Bashkirs, Crimean Tatars and a number of the northern Caucasus Turkic speakers, have to a larger or lesser extent, preserved their identity, even after many political transformations.

Lipka Tatars, or Lipkas, are an ethnic and religious Sunni-Muslim minority in Belarus, Lithuania and Poland, accounting for about twelve thousand people. The majority of Lipkas — about seven thousand people reside nowadays in Belarus (National Statistical Committee of the Republic of Belarus. Population Census of the Republic of Belarus 2009. Available at: http://belstat.gov.by/homep/ru/perepic/2009/main_new.php. (Accessed: 18th June 2014)) and are below referred to as Belarusian Lipka Tatars (BLT). Lipkas are thought to descend from migrants of the Golden Horde and post-Golden Horde khanates^3^. According to historical records, they were invited as mercenaries to the territory of the Grand Duchy of Lithuania during the period from the late 13^th^ to the middle of the 16^th^ century. The core of Lipkas as a distinct ethnic group had formed already by the end of the 14^th^ century^3^ and it is likely that the documented migrations from the Crimea, Azov Sea and Don River regions played a major role in this process^4^,^5^,^6^. Initially, these migrants spoke one or several languages belonging to the Kipchak group of the Turkic languages^7^, but by the beginning of the 17^th^ century they had already switched to Belarusian or Polish^5^. This relatively rapid language replacement may have been facilitated by (i) the initial linguistic heterogeneity of Lipka’s ancestors, (ii) the widespread practice of marriages with locals during the 14^th^ and 15^th^ centuries, and (iii) participation of Lipkas in the military service of the host country^8^. Despite these aspects of cultural integration into East European society, Lipkas retained their Muslim religion, which remains a core component of their ethnic self-identification today. The practice of marriages with locals was subsequently banned, helping to establish and maintain the ethnic boundaries between Lipkas and their neighbours^5^,^8^. The transition from a nomadic to sedentary lifestyle, language change, and their preservation of the Muslim religion, are the three features that make Lipkas an intriguing population not only *per se* but also in the broader context of the population history of the Eurasian Steppe.

We have shown previously^9^ that BLT bear about 30% of East Eurasian mitochondrial DNA (mtDNA) haplogroups, while the rest of their mtDNA pool can be broadly defined as West Eurasian in origin, with some haplotypes shared between BLT and Belarusians. Our preliminary Y-chromosomal DNA analysis revealed considerable heterogeneity of the BLT patrilineal heritage, witnessed by the presence of haplogroups typical of East European, Caucasian, Volga-Uralic, Central Asian and Siberian populations, with some Y-STR haplotypes shared specifically between BLT and Belarusians^10^.

Here, in order to infer the origin of different components of the gene pool of the present-day BLT, together with the mode and timing of their admixture in the past, we analyze BLT samples in the context of 120 complete mtDNA sequences from 35 Eurasian populations including 11 BLT and 27 new sequences from other populations, 1628 Y-STR haplotypes from 81 populations including 74 BLT and 80 new haplotypes from other populations, and 1231 genome-wide genotypes from 87 populations including six new BLT samples.

## Results

### Y-chromosomal variation

**Figure 1:**
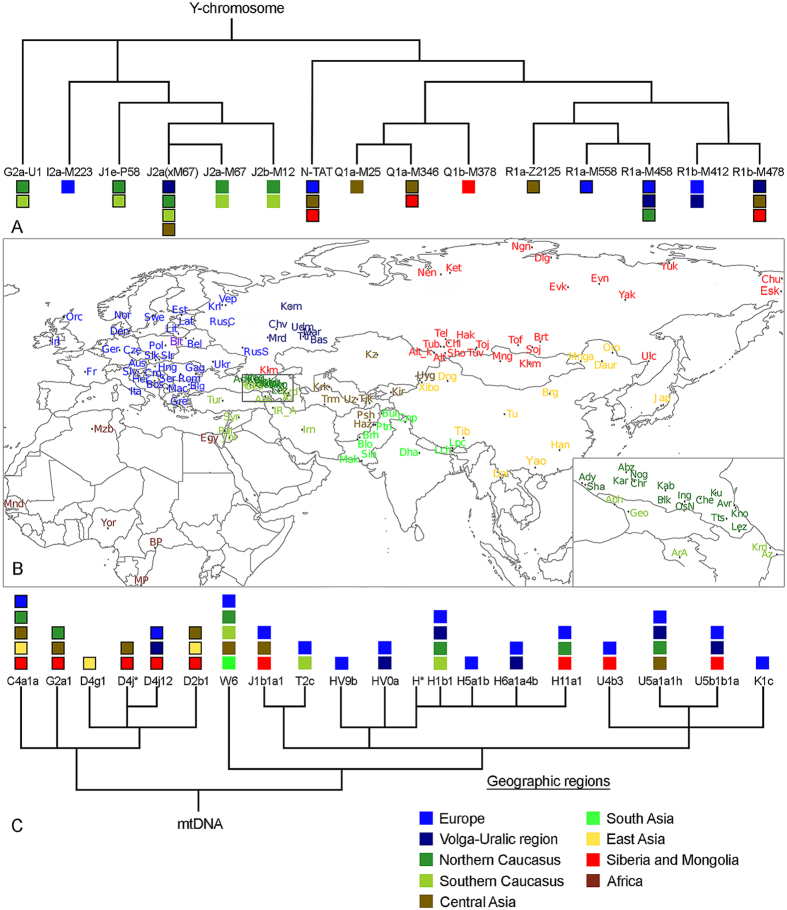
(**A**) Schematic phylogeny of the Y-chromosome tree in Belarusian Lipka Tatars (updated from^10^). (**B**) Geographic map showing population background used in the study. Belarusian Lipka Tatars are indicated in purple. Supplementary Table 1 lists population names that correspond to the abbreviations in the map, and which populations were used in Y-chromosome, mtDNA or autosomal SNP analyses. Caucasus region is zoomed-in and shown in bottom right corner of the map. Map was created in R v3.1.1 using “maps” and “mapproj” packages (R: A Language and Environment for Statistical Computing, R Core Team, R Foundation for Statistical Computing, Vienna, Austria (2016) https://www.R-project.org”). (**C**) Schematic phylogeny of the mtDNA tree in Belarusian Lipka Tatars (updated from^9^). Colored squares at the tree tips indicate geographic regions where same Y-chromosome and mtDNA haplogroups occur nowadays. Squares with black borders indicate that phylogenetically close haplotypes between Belarusian Lipka Tatars and other populations were detected based on phylogenetic analysis of complete mtDNA sequences or Y-STR haplotypes carried out in this study; open squares summarize information on phylogeographic distribution of respective haplogroups (for full list of references see Supplementary Information Text (Full List of References for Fig. 1)). For paraphyletic mtDNA haplogroup H* no data is provided as it may correspond to different H subclades with broad geographic distribution.

The map in Fig. 1B outlines the geographic background of populations used in this study (population abbreviations are explained in Supplementary Table 1). The backbone of the Y-chromosome phylogenetic tree of BLT is shown in Fig. 1A; detailed tree with haplogroup frequencies present among BLT is presented in Supplementary Fig. 1 (see also Supplementary Information Text (Genetics)). The BLT patrilineal gene pool comprises 15 haplogroups, some with clear geographical affinities, including East Europe and the Volga-Uralic region (N-Tat, R1a-M458, R1a-M558, R1b-M412 and R1b-M478), Central Asia (R1a-Z2125 and Q-M242), South Siberia (Q-M242 and R1b-M478), the Caucasus and the Middle East (G2a-U1, J1-P58, J2a-M410 and J2b-M12). Although the haplogroup compositions overlap substantially between BLT and Belarusians, frequencies of some lineages such as I-P37, J-M172, Q-M242, R-Z2125 and R-M558, differ significantly between the two populations (Supplementary Table 2)^11^.

In order to further elucidate the relationships between BLT and other Eurasian populations, we determined 17-loci Y-STR haplotypes for 74 BLT samples and 80 samples from other populations (Supplementary Table 3), constructing median-joining networks for haplogroups G2a-U1, J1-P58, J2a(xM67), N-Tat, Q1a-M346, R1a-Z2125, R1a-M458, R1a-M558, and R1b-M478, including published haplotypes (Supplementary Figs 2–10, Supplementary Table 4). We found that Q1a-M346, R1b-M478 and R1a-Z2125 Y-STR haplotypes are most similar phylogenetically between BLT and Turkic-speaking populations of Central Asia (Kyrgyz, Kazakhs and Uzbeks) and South Siberia (Tuvinians, Khakassians and Shors) (Supplementary Figs 6,9 and 10, Supplementary Tables 5 and 6), whereas G2a-U1 and J1-P58 haplotypes of BLT are the closest in their phylogeny to those from Caucasus populations (Supplementary Figs 2 and 3, Supplementary Tables 7 and 8). Note that although R1b-M478 has the highest frequency in Bashkirs^12^, their Y-STR-haplotypes differ from those found in BLT. In particular, Bashkirs have 19 repeats in the DYS390 locus, while BLT have 21–22 repeats. Haplotypes of J2a(xM67) found among BLT are similar to those from populations of Central Asia, North Caucasus, Iran and Volga-Uralic region (Supplementary Fig. 4, Supplementary Table 9). Thus, the paternal gene pool of BLT incorporates various haplotypes that are widely spread nowadays across Central Asia, South Siberia, Volga-Uralic region and the Caucasus. On the other hand, the presence of haplogroups R1a-M458 and R1a-M558 suggests genetic admixture within Europe as there are haplotypes shared between BLT and Central-East Europeans (Belarusians, Ukrainians, Poles, Russians etc.) (Supplementary Figs 7 and 8, Supplementary Tables 10 and 11). Although some of these European haplotypes (R1a-M458) are also found among Turkic-speaking Nogais, Karanogais and Volga Tatars (Supplementary Fig. 7), their flow into BLT gene pool from the host populations seems to be more plausible considering the phylogeographic spread patterns of those haplogroups^13^. Interestingly, the two N-Tat haplotypes found in BLT may originate from different sources: one is similar to variants spread in Central Asian and Siberian populations, whilst the second is typical of East European populations (Supplementary Fig. 5).

### Mitochondrial DNA variation

The backbone of the mtDNA phylogenetic tree for BLT is shown in Fig. 1C. In our previous study on the maternal gene pool of BLT^9^ (see also Supplementary Information Text (Genetics)) we found that 26 out of 80 individuals had mtDNA lineages belonging to haplogroups C, G and D (Supplementary Table 12), whose current frequencies of distribution suggest that they can be considered as East Eurasian^14^. Here, in order to further elucidate matrilineal gene flow marked by East Eurasian-specific lineages, we have generated complete mtDNA sequences for 27 individuals from 13 Eurasian populations belonging to haplogroups C4a1’5, G2a1, D4j*, D4j12, D4g1 and D2b1, whose HVSI sequences are identical or similar (no more than 3 additional substitutions) to those found among BLT. High resolution phylogenetic trees show that C4a1 haplotypes of BLT (Blt_10 and Blt_11) cluster together with a Kazakh sequence (Supplementary Fig. 11); one D4j* sample of BLT (Blt_4) forms an individual branch together with Kyrgyz and Teleut samples (Supplementary Fig. 11); Blt_3 and Blt_7 together with Barga Mongol and Volga Tatar sequences form a new branch of the D4j12 sub-haplogroup (Supplementary Fig. 11). The only D4g1 sample of BLT (Blt_8) belongs to a “Japanese” cluster, but it has six additional substitutions (Supplementary Fig. 11). D2b1 BLT sample Blt_9 joins a group that includes sequences from Siberian, East and Central Asian populations: Han, Tibetan, Kazakh, Yakut, Evenk, Buryat, Khamnigan and Kalmyk (Supplementary Fig. 11). Finally, the three G2a1 BLT sequences Blt_1, Blt_2 and Blt_6 cluster together with Tuvinians, Karachai, Kyrgyz, Buryat and Yakut samples (Supplementary Fig. 11). Taken together, our data show that BLT share part of their matrilineal ancestry with Central Asian, Siberian and East Asian populations.

### Belarusian Lipka Tatars in a Eurasian genetic context according to whole genome SNP variation

**Figure 2:**
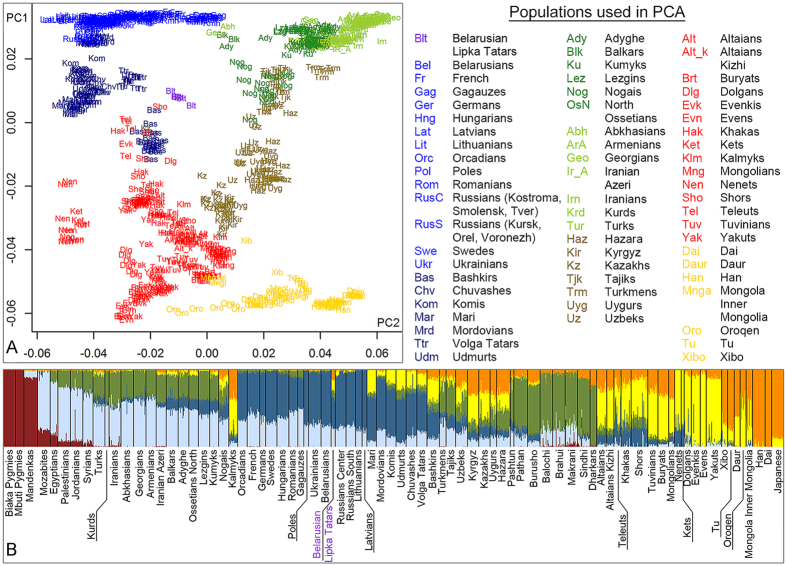
(**A**) PC plot PC1 *vs* PC2 based on whole genome SNP variation in 63 Eurasian populations. PC1 = 3.8; PC2 = 0.6. (**B**) k6 ADMIXTURE plot showing genetic structure of Belarusian Lipka Tatars in the background of 81 world populations. Genetic structure of populations at k2-k10 is shown in Supplementary Fig. 18.

Results of the principal components analysis^15^ are presented in the PC plot in Fig. 2A that shows the place of BLT on a Eurasian genetic variation map. In the plot of PC1*vs*PC2, PC1 separates populations along a west-east axis, placing European populations in the top left-hand corner, whilst East Asian and Siberian populations are located towards the bottom of the plot (Fig. 2A). PC2 separates the populations along a north-south axis, placing North-East Europeans and South Caucasus/Middle Easterners opposite to each other (Fig. 2A). BLT form a dense group that falls between Central-East European and Central Asian groups.

Average population pairwise distances reveal significant difference between BLT and Belarusians as well as with other East Europeans (F_ST_ = 0.014) (Fig. 2A, Supplementary Table 13). On the other hand, the smallest genetic distances are those between BLT and Volga Tatars, Nogais from North Caucasus, Tadjiks and Uzbeks from Central Asia (F_ST_ = 0.007) (Supplementary Table 13).

Next, a clustering algorithm ADMIXTURE^16^ was used to identify potential ancestral components among the genomes of BLT. Considering the model with six ancestral populations (Methods section provides details on choosing the number of k; Supplementary Table 14 lists populations used), around two-thirds of the BLT genomes are composed of the “European” (blue) and “Middle Eastern/Caucasus” (light blue) components (Fig. 2B). The remaining one-third belongs to two sub-variants of the general East Eurasian component: orange, typical for Han population, and yellow, which is well represented in Siberian populations. Taken together, both PC and ADMIXTURE analyses suggest the presence of a significant amount of East Eurasian-specific alleles among the autosomal genomes of BLT.

To provide a formal test for admixture in BLT, we performed the three population test^17^ specifying various Eurasian populations (Supplementary Table 14) as potential sources. In Supplementary Table 15 we report only those combinations of sources that produced statistically significant negative f3-statistics (z-score < −1.64), thereby supporting the scenario of admixture. The lowest f3 values are revealed for European populations on one side and Chinese populations and East Siberian Evens on the other (Supplementary Table 15). Overall, the f3 test results are significant for BLT when one source of admixture is represented by Europeans and the second source includes Siberian, Caucasus, Central and East Asian populations (Supplementary Table 15).

### Inferring sources and dates of admixture in Belarusian Lipka Tatars

We used ChromoPainter together with the fineSTRUCTURE clustering algorithm^18^ to classify individuals into groups using information on shared extended genomic haplotypes. The output of fineSTRUCTURE was used with GLOBETROTTER^19^ to deduce sources of admixture, quantify their fractions and to date admixture events in BLT.

The fineSTRUCTURE dendrogram is shown in Supplementary Fig. 12; it is also represented together with the ChromoPainter chunkcount coancestry matrix (Supplementary Fig. 13) and Pairwise coincidence matrix (Supplementary Fig. 14). The dendrogram (Supplementary Fig. 12) has four major clusters: two include mostly Chinese/Siberian and Siberian/Central Asian populations, one encompasses European/Caucasus/Middle Eastern populations, and the fourth one includes populations from the Volga-Uralic region/Central Asia. All six BLT individuals form a single branch within the Volga-Uralic region/Central Asian cluster, where they group together with Bashkirs as well as with some individuals from Central Asia/Siberia (i.e. Shor/Teleut/Khakas/Kazakh individuals). It should be noted that a substantial share of European-like ancestry in genomes of those Central Asian and South Siberian individuals (Fig. 2A,B) indicates a history of admixture. Collectively, haplotype-based clustering analysis suggests that a genetic profile similar to the one observed in BLT might result from a mixture of European-like and South Siberian/Central Asian-like ancestry.

Two “best-guess” sources of admixture — Mongola-like (30%) and Hungarian-like (70%) – were inferred for BLT using the GLOBETROTTER algorithm (Supplementary Table 16; Supplementary Table 17 lists genetic clusters used in the analysis; Supplementary Fig. 15 provides examples of LD curves). Around 80% of the Hungarian-like source is composed of East Slavic, Armenian and Lithuanian/Latvian genetic groups. The Mongola-like source is represented largely by genetic groups from South Siberia and North China (namely, Mongola/Xibo, Han and Mongolian/Kalmyk groups) (Supplementary Table 16). Assuming a single episode of admixture between the two sources, this is dated to about 22 generations ago, which equates to the 13^th^ century (12–14 centuries) assuming a generation time of 28 years (Supplementary Table 16).

Unlike GLOBETROTTER, which uses genetically related groups of individuals and haplotype information to model sources and dates of admixture, the ALDER algorithm uses groups of individuals labeled according to their ethnicity/geography and information from individual SNPs^20^. To test whether this difference affects the inference, we applied the ALDER algorithm to derive sources and dates of admixture based on LD decay in genomes of BLT and a range of Eurasian populations (Supplementary Table 14). According to the ALDER results, “West Eurasian” sources include European (French, Latvian) and Caucasus populations (Abkhazians, Georgians and Kurds from South Caucasus), whereas “East Eurasian” sources are related to populations of Siberia, Mongolia and East Asia, which is in broad agreement with the GLOBETROTTER results (Supplementary Table 18, Supplementary Table 16). Inferred dates of admixture — around 26 (+/−6) generations ago (or 11−15 cc AD assuming 28 years per generation) – are also in accord with the results of the GLOBETROTTER analysis (Supplementary Table 16).

### Genomic IBD segments shared by Belarusian Lipka Tatars and Eurasian populations

**Figure 3:**
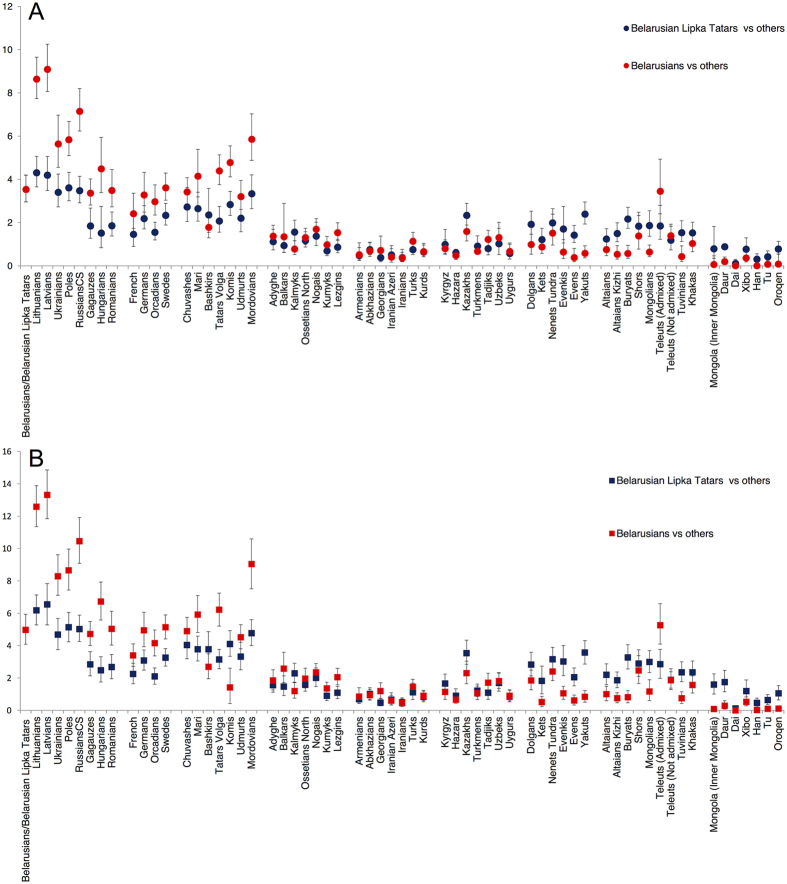
Parameters of IBD sharing between Belarusian Lipka Tatars, Belarusians and other Eurasian populations. y-axes indicate: (**A**) average number of IBD segments per pair of individuals for each combination of populations analyzed; (**B**) average total length of genome shared identical by descent (in cM) per pair of individuals for each combination of populations analyzed. See Supplementary Table 14 for note about the Teleut samples.

We assessed patterns of IBD sharing between BLT and European, Caucasus/Middle Eastern, Central Asian, Siberian/Mongolian and Chinese populations using a refined IBD algorithm^21^,^22^, and compared them with the IBD sharing between Belarusians and the same groups of populations (Fig. 3). Based on limited inference from haploid data^11^ we assumed Belarusians to be a good proxy for differentiating between a background, hence geography determined IBD sharing and IBD sharing due to recent migration from Siberia, Central and East Asia.

BLT demonstrate the highest level of IBD sharing with Central-East European host populations, followed by populations of the Volga-Uralic region (~4 and 2.6 IBD segments (5 and 4 cM) per pair, respectively) (Fig. 3). The degree of IBD sharing between both BLT and Belarusians on the one hand and populations to the south — North and South Caucasus — on the other, reduces abruptly (~1 and 0.6 segments (1.6 and 0.8 cM) per pair, respectively (Fig. 3). In contrast to this pattern, BLT display increased level of average number of IBD segments and average total length of genome shared identical-by-descent with Kazakhs from Central Asia (2 segments and 3.5 cM per pair), and most of the Siberian and Mongolian populations (~1.7 segments (2.5 cM) per pair), compared to Belarusians, who on average share 1 IBD segment (1.6 cM) per pair with individuals from those populations (Fig. 3). Thus, IBD analysis reveals pronounced admixture between BLT and their contemporary host populations on one hand, and a signal of shared genetic ancestry with populations from a region spanning Kazakhstan, South Siberia/Mongolia and northern China on the other.

### Runs of homozygosity (RoH)

A relatively high proportion of the East Eurasian component in the gene pool of BLT, around 30% (Fig. 2B; Supplementary Table 16), could have been retained due to endogamy and drift, as BLT have been partially isolated from their host populations^4^. To test this hypothesis, we assessed RoH in genomes of BLT, Belarusians and other Eurasian populations (populations listed in Supplementary Table 14). Indeed, we found that the total number and length of homozygous segments in BLT are higher than in Belarusians, and are comparable to those in South Siberian populations (e.g., Altaians, Buryats and Tuvinians), which are characterized by lower population density, smaller effective population sizes (N_e_) and high rates of endogamy^23^ (Supplementary Fig. 16).

## Discussion

In contrast to their immediate neighbors, such as Belarusians, Poles and Lithuanians, the gene pool of BLT, alongside the dominant West Eurasian component bears a relatively large — about 30% — East Eurasian component (Fig. 1A,C, Supplementary Figs 2–12, Fig. 2A,B, Supplementary Tables 15 and 16). Partial isolation of BLT from the host population due to ethnic, in particular religious, differences, during the lengthy period of their joint residence, have likely contributed to a preservation of this East Eurasian ancestry in the genomes of Lipkas.

### Likely sources of East Eurasian ancestry in the gene pool of Belarusian Lipka Tatars

Among the two major genetic components revealed in the gene pools of BLT, an East Eurasian one, relating BLT to the Siberian/Mongolian region, potentially incorporates information about their nomadic ancestry. Therefore, we made an in-depth characterization of the East Eurasian component in the gene pool of BLT using three sets of data: Y-chromosome, mtDNA and genome-wide genotypes.

The paternal Y-STR haplotypes of J2a(xM67), Q1a-M346, R1a-Z2125 and R1b-M478 (Supplementary Figs 4, 6 and 9, Supplementary Table 4), as well as complete mtDNA sequences of haplogroups D4j*, D4j12, D2b1 and G2a1 (Supplementary Fig. 11) from the BLT, are phylogenetically closest to those found predominantly among modern Central Asian (Kazakhs, Kyrgyz, but also Uzbeks) and Siberian/Mongolian populations (mainly Buryats, Tuvinians, Khakasses and Teleuts, but also Shors, Barga Mongols, Kalmyks, Khamnigans, Yakuts and Evenkis). Genetic links between BLT and Caucasus and, to a lesser extent, Volga-Uralic populations, are exemplified by mtDNA haplogroups D4j12 and G2a1, and Y-chromosome haplogroups J2a(xM67), G2a-U1 and J1-P58 (Supplementary Figs 11,4,2,3 and 10). The distribution of IBD segments and autosomal haplotypes demonstrate a strong affinity between BLT and populations from South Siberian/Mongolian region, and with Kazakhs (Fig. 3; Supplementary Table 16). As the BLT uniparental haplotypes are generally absent in their neighboring East Europeans, including Belarusians^11^,^24^ and as there is an excess of IBD segments between geographically distant populations of BLT and Siberians/Mongolians, we conclude that the presence of these haplotypes in the BLT gene pool is a result of a migration event(s) rather than a long-term process of genetic diffusion. Moreover, as BLT share East Eurasian-like haplotypes with various modern populations across Eurasia from the Caucasus to North-East China, it is likely that complex migration/admixture events, involving highly mobile ancestral population(s) have contributed to the formation of the BLT gene pool. Another noteworthy conclusion from our data is that whatever migration event(s) brought East Eurasian genetic components to the gene pool of modern BLT, it has involved both men and women.

### West Eurasian component in the gene pool of Belarusian Lipka Tatars

Based on what is known of the Eurasian Steppe nomads and BLT from historical records, as well as from previous genetic studies, one can assume that the West Eurasian admixture evident in the gene pools of the ancestors of BLT increased gradually during their history. Here, we should consider at least three possible steps: a) ancient admixture in Central Asia and southwest Siberia and Mongolia, which is supported by the presence of the West Eurasian component in the gene pools of both modern and ancient populations of this area^25^,^26^,^27^,^28^; b) admixture during the historic migrations of nomadic populations in the territory of the Pontocaspian Steppe^2^,^8^ and c) admixture events during and after their settlements in the territory of the Grand Duchy of Lithuania, i.e. after becoming known as Lipkas.

The idea of gene flow from the host populations (c) to the settled Lipkas is supported by our previous mtDNA data^9^ as well as by documented marriages between ancestors of BLT and local women during their early settlement in the Grand Duchy of Lithuania^8^,^29^,^30^. Similarly, IBD analysis from this study shows that BLT share the highest number of common genetic segments with populations from East Europe (Fig. 3). We also see a close resemblance of Y-STR haplotypes of haplogroup R1a-M458 between BLT and Belarusians and/or Poles, suggesting a male mediated gene flow from the host populations to BLT (Supplementary Fig. 7, Supplementary Table 4). Although R1a-M458, frequent in Central and East Europe^31^, is also found at low frequencies elsewhere, including Nogais, Karanogais and Shapsugs as well as Volga Tatars (Supplementary Fig. 7, Supplementary Table 4)^32^,^33^, it is more parsimonious to assume that BLT acquired this haplogroup when already settled in East Europe.

The likely admixture during step (b) is demonstrated by the presence in BLT Y-chromosome haplogroups G2a-U1, J1-P58 and J2a(xM67), typically found in the Caucasus (Supplementary Figs 2–4, Supplementary Table 4). This is also inferred by the GLOBETROTTER analysis, in which the major –“Hungarian” – source of admixture in BLT contains around 20% of an Armenian-like genetic component (Supplementary Table 16). Some of the mtDNA haplogroups found in BLT, e.g. W6, may have also arrived from the Caucasus^34^ because the Golden Horde spanned well into this region^35^. It is possible, therefore, that the ancestors of BLT already had some, or even substantial, European and Caucasian genetic legacy before they settled in the territory of the Grand Duchy of Lithuania.

Our analyses suggest admixture for BLT that took place within 12–14 centuries AD (Supplementary Tables 16 and 18). It should be noted, however, that in the case of several subsequent admixture events, spread over a wider time window — a likely case for nomads — these analytical approaches tend to give dates corresponding to the dominant, often later steps of the admixture process^2^,^19^.

### Belarusian Lipka Tatars as a former Turkic-speaking population

Although BLT today speak Belarusian or Russian, it is documented that their ancestors spoke a Kipchak language(s) of the Turkic family but switched to Slavic sometime after their settlement in the territory of the Grand Duchy of Lithuania (Supplementary Information Text (Linguistics))^5^,^7^,^36^. Furthermore, it is interesting to note that several tribal names in BLT are found simultaneously in numerous contemporary Turkic- and Mongolian-speaking peoples, suggesting that the same Turkic and initially Mongolian tribes contributed to the ethnogenesis of these populations including BLT (Supplementary Table 19). Thus, both linguistic and anthroponymic evidence suggest a cultural affiliation of BLT with many Turkic-speaking populations living today across the Eurasian Steppe.

Many Turkic-speaking populations, whilst genetically resembling their non-Turkic geographic neighbors, have retained genomic chunks shared with populations of South Siberia and Mongolia (SSM)^2^. Likewise, here we have found an excess of IBD segments shared between BLT and Siberian/Mongolian/northern Chinese populations, as well as Kazakhs from Central Asia, when compared to Belarusians (Fig. 3). We suggest, that the IBD pattern observed in BLT, currently non-Turkic speakers, reveals a “Turkic-specific” genetic signal shared to some extent by almost all modern Turkic speakers^2^.

The proportion of the presumed East Eurasian component that is likely to incorporate this “Turkic-specific” genetic footprint in the genomes of BLT, is substantially higher (~30%) when compared to many Turkic-speaking populations in western Eurasia such as Gagauz, Turks, Iranian Azeri, Balkars, Kumyks and Turkmens, and is as high as in the Volga Tatars according to (Fig. 2B). In this context it is interesting to compare BLT with Gagauz people, who also reside in the western fringes of Eastern Europe and, similarly to BLT, are thought to originate from Medieval Turkic nomads, either from the “Russian Steppe” or migrants from Anatolia^37^. In contrast to BLT, however, although Gagauz switched from Islam to Orthodox Christianity in medieval times, they still speak a language close to Oghuz Turkic spoken in Turkey. Furthermore, the uniparental gene pools of Gagauz harbor no haplogroups that can be unanimously described as East Eurasian^38^,^39^; and they virtually lack an East Eurasian signal in their autosomal genomes^2^, confirmed in the present study (Figs 2 and 3). Hence, peoples of two mid-European Turkic enclaves must have had contrasting demographic histories; while BLT retained a strong genetic signal of their nomadic, in part East Eurasian, origin, in the case of Gagauz a language shift among a Medieval Balkan population to Turkic is a more likely scenario.

## Conclusions

We have characterized the genomes of Belarusian Lipka Tatars, an ethnic community living in Eastern Europe for longer than a half a millennium. Lipkas are unique in several ways; preserving their Sunni-Muslim faith in a Christian surrounding, they nevertheless, already many centuries ago, became a Slavic-speaking community. About two thirds of their autosomal, as well as haploid patrilineal and matrilineal heritage can be best described as West Eurasian, including a minor input from the Caucasus area, while remaining third of their genetic heritage derives from East Eurasia. The latter suggests that Lipka descend not solely from light cavalry mercenaries hired by the Great Duchy of Lithuania, but likely also from nomadic warriors, who had arrived and settled down in central-east Europe as families. An overlap of tribal names preserved in the social memory of BLT, with those present in many current Turkic-speaking populations as well as in Mongols, complements conclusions drawn from our genetic analyses, and is in accord with a view that the Golden Horde was a confederation of tribes of different ethnic origins.

## Material and Methods

### Sampling

DNA samples of BLT used for Y-chromosome (N = 74) and mtDNA (N = 80) genotyping are from a previous study^9^,^10^. Sampling locations of BLT are shown in Supplementary Fig. 17. In addition, 80 samples from various populations were used for Y-STR genotyping and 38 samples including BLT were used for complete mtDNA sequencing (see Supplementary Table 20 for details about samples). DNA samples for Illumina genome-wide genotyping were collected for this study from six BLTs. In all cases, genomic DNA was extracted from venous blood of unrelated individuals following a standard phenol/chlorophorm procedure^40^.

### Ethics Statement

The samples analyzed in the study were collected after having obtained a written informed consent from all donors. The project was carried out in accordance with the approved guidelines by the Bioethics Committee of the Belarusian State Medical University (Minsk, Belarus), the Research Ethics Committee of the University of Tartu. All experimental protocols were also approved by the Bioethics Committee of the Belarusian State Medical University (Minsk, Belarus), the Research Ethics Committee of the University of Tartu (UT 225/T-9).

### Uniparental data: genotyping

Y-chromosome haplogroups for 74 BLT were determined in a previous study^10^. Samples belonging to haplogroup G-P15 were additionally genotyped for markers P303 and U1, while R1a(xM458) samples were further genotyped for markers Z93, Z2125, Z282 and M558. Details of genotyping are provided in Supplementary Table 21. Current Y-chromosome phylogeny^41^ and nomenclature were used.

All 74 samples were previously genotyped for 17 Y-STR loci using AmpFLSTR® Yfiler® kit^10^. In this study additionally 22 new Y-STR haplotypes for J1-P58, 47 new Y-STR haplotypes for J2a(M67), 11 new Y-STR haplotypes for R1a-M458 from various populations were generated (Supplementary Table 3).

MtDNA haplogroups for 80 unrelated BLT individuals as well as complete mtDNA sequences for 11 BLT were determined in^9^. Haplogroups were assigned according to the phylogeny present on www.phylotree.org build17 (18Feb2016)^42^.

MtDNA was sequenced as described in either^43^ or^44^ using an ABI 3500xL Genetic Analyzer for 27 individuals belonging to haplogroups C4a1, D2b1, D4j and G2a1 from various populations (Supplementary Table 20). For sequence alignment and analysis ChromasPro 1.7.1.0 or SeqScape 2.5 (Applied Biosystems) were used. Substitutions were recorded relative to rCRS^45^ and RSRS^46^ (Supplementary Table 22). FASTmtDNA utility (www.mtdnacommunity.org) was used to convert sequences from rCRS-format to RSRS-format. Complete mtDNA sequences generated in this study are available at the NCBI (GenBank accession numbers KX358471-KX358508).

### Whole genome SNP data

Six BLT were genotyped on Illumina 730k platform and analyzed together with 1238 individuals from 83 Eurasian populations from previous studies (Supplementary Table 14). The dataset was preprocessed with PLINK v1.07^47^ to ensure that SNPs on the 22 autosomes with minor allele frequency >1% and missing genotype rate <3% are included and individuals with missing genotype data >3% are omitted. Relatives of the first and the second degree (one individual from a detected pair) were also removed^48^. Because some analyses required that SNPs are in linkage equilibrium, the SNPs with r^2^ > 0.4 were pruned out in windows 200 SNPs with step 25 SNPs. Exact numbers of individuals, populations and SNPs used in each analysis are specified in Supplementary Table 14. Possible limitations of using six BLT samples in the genome-wide analyses are discussed in Supplementary Information Text (Material and Methods). The genome-wide genotypes of six BLTs are available at Gene Expression Omnibus (NCBI GEO accession number GSE82309) as well as in PLINK format in our website at www.ebc.ee/free_data.

### Whole genome SNP data analyses

#### Runs of homozygosity

Runs of homozygosity (RoH) in BLT were called using PLINK^47^. RoH were defined as regions with at least 50 consecutive SNPs in a window of 1500 kb with a gap less than 1000 kb between adjacent regions, with density of SNP coverage within the region no more than 1 SNP per 50 kb, and with no more than five missing calls and one heterozygote per window^49^,^50^. As total length of RoH segments varied substantially among individuals, we first took natural logarithm of these values for each individual and then calculated the mean for each population sample. We also calculated mean number of RoH segments for each population sample.

#### F_ST_

Mean population pairwise F_ST_ were calculated for BLT and a range of Eurasian populations (Supplementary Table 14) as in^51^.

#### PCA

PC analysis was performed using *smartpca* software implemented in EIGENSOFT package^15^. Populations used in the analysis are listed in Supplementary Table 14. The genetic structure of populations was inspected at first five PC; the most informative first two were discussed in the main text.

#### ADMIXTURE

Model-based clustering algorithm ADMIXTURE^16^ was used to infer genomic ancestral components in BLT in a worldwide context (Supplementary Table 14). We tested 2 to 10 ancestral clusters (k) (Supplementary Fig. 18). Calculations for each k were repeated 100 times, convergence between runs was assessed using log-likelihood scores (LL) as in^52^.

#### f3 test

f3(target; source1, source2) – is a phylogeny based formal test whether a target population is related to source populations through admixture^17^. Negative values of f3 statistics with z-score below −1.64 indicate statistically significant admixture in a target population with populations related to the specified sources.

### Analysis of segments identical by descent (IBD)

Refined IBD algorithm implemented in BEAGLE v4.0^21^,^22^ was used to detect IBD segments shared by BLT and their host population (Belarusians) with a set of Eurasian populations. Sample sizes for all populations were balanced: ~5–6 individuals per each population (Supplementary Table 14). Refined IBD was run with the default settings except the *ibdtrim* parameter, which was reduced to 20 markers because of lower marker density in our dataset. IBD segments longer than 1 centiMorgan (cM) were analyzed. Two parameters that characterize IBD sharing were estimated. (i) An average number of IBD segments per pair of individuals for each BLT-[*population*] and Belarusians-[*population*] pair. (ii) An average total length of genome shared identical-by-descent per pair of individuals for the same BLT-[*population*] and Belarusians-[*population*] combinations. 95% CI for the average number of IBD segments were calculated as in^53^, while CIs for the average total length of genome shared identical-by-descent were calculated using the bootstrap percentile method with 10000 replicates.

### fineSTRUCTURE, GLOBETROTTER

SHAPEIT v2.r790 was used to phase autosomal SNP data^54^. SNPs in phased haplotypes were subsequently imputed with IMPUTE2 software^55^ using 1000 Genomes Project sequence data as a reference. Genetic maps were taken from the same 1000 Genomes download with physical positions in NCBI b37 coordinates. All SNPs in the data, which were absent in the reference panel, were excluded. The “chromosome painting” was performed using the ChromoPainter v2^18^,^19^. Prior to the main ChromoPainter analysis, we have analyzed a subset of the data with 10 EM iterations to calculate the recombination scaling constant (Ne) and mutation probabilities. For the fineSTRUCTURE analysis every individual was “painted” using every other individual^18^. In the fineSTRUCTURE v0.0.2 MCMC analysis was performed using 10000000 MCMC iterations and 5000000 burn-in iterations keeping every 10000 sample. The tree was built using the obtained population assignments with the 10000000 iterations for hill-climbing and 100000000 maximum number of tree comparisons for splitting/merging. For the GLOBETROTTER analysis^19^ individuals were merged into 88 genetic groups according to the structure inferred by fineSTRUCTURE. Thereafter individuals were “painted” using inferred genetic groups; the group represented by BLT was not used as a donor group. Genetic groups with less than five individuals were also not used as was proposed in^56^ (except Kets as there are only four Ket individuals in the data set, who formed a single genetic group). In the end, 60 admixture donor groups were used (Supplementary Table 17). To define admixture dates and proportions two analyses were performed, one of which was standardized by a “NULL” individual when performing inference. To obtain confidence intervals, 100 bootstrap re-samples were used for both types of analyses.

### ALDER

ALDER^20^ was applied to BLT and a range of Eurasian reference populations (Supplementary Table 14) first, to test for the presence of admixture, and then to date this admixture. Results of ALDER are reported in Supplementary Table 18.

### Uniparental markers analyses

Y-STR phylogenetic networks were constructed using the Network software version 4.6.1.2, applying the median-joining algorithm^57^ (Fluxus Technology Ltd, http://fluxustechnology.com) and drawn using Network Publisher. Weighting of Y-STR loci for each haplogroup was done as described in^58^. Datasets of Y-STR haplotypes for each haplogroup were compiled individually so that populations included maximally represent the phylogeographic pattern of a respective haplogroup. The final number of Y-STR loci used for network analyses for each haplogroup depended on the STR sets available in the literature (Supplementary Table S4). For better visualization, minimal networks including only haplotypes no more than three or in some cases two mutation steps from BLT were built and shown in the figures alongside with the complete networks. Arlequin v3.5.2.2^59^ was used to calculate population pairwise R_ST_ values for Y-chromosome haplogroups G2a-U1, R1a-M458, J2a(xM67), R1a-M558, R1a-Z2125, J1-P58 and Q1a-M346 (Supplementary Tables 5–11); two haplogroups (N-Tat and R1b-M478) were left out of the R_ST_ calculations due to small number of BLT haplotypes affiliated to those two haplogroups.

Complete mtDNA sequences were incorporated into previously published phylogenetic trees: phylotree.org^42^ for G2a1 and ref. 60 in the case of haplogroups C4a1 and different subgroups of haplogroup D using maximum parsimony approach.

## Additional Information

**How to cite this article**: Pankratov, V. *et al*. East Eurasian ancestry in the middle of Europe: genetic footprints of Steppe nomads in the genomes of Belarusian Lipka Tatars. *Sci. Rep.* **6**, 30197; doi: 10.1038/srep30197 (2016).

## Supplementary Material

Supplementary Information

Supplementary Table S1

Supplementary Table S2

Supplementary Table S3
