# Supplementary material for: East Eurasian ancestry in the middle of Europe: genetic footprints of Steppe nomads in the genomes of Belarusian Lipka Tatars
Source: Sci Rep. 2016 Jul 25;6:30197. doi: 10.1038/srep30197 (PMC4958967; doi:10.1038/srep30197)

Supplementary Information

East Eurasian ancestry in the middle of Europe: genetic footprints of Steppe nomads in the genomes of Belarusian Lipka Tatars

Vasili Pankratov[1]#, Sergei Litvinov[2,3], Alexei Kassian[4,5], Dzmitry Shulhin[6], Lieve Tchebotarev[7], Bayazit Yunusbayev[3], Märt Möls[8], Hovhannes Sahakyan[3,9], Levon Yepiskoposyan[9], Siiri Rootsi[3], Ene Metspalu[3,10], Maria Golubenko[11], Natalia Ekomasova[12], Farida Akhatova[12,13], Elza Khusnutdinova[2,12], Evelyne Heyer[14], Phillip Endicott[14], Miroslava Derenko[15], Boris Malyarchuk[15], Mait Metspalu[3], Oleg Davydenko[1], Richard Villems[3,10]#, Alena Kushniarevich[1,3]#

# Correspondence to vasilipankratov@gmail.com, lkushniarevich@gmail.com, rvillems@ebc.ee

Authors affiliations:

[1]Institute of Genetics and Cytology, National Academy of Sciences of Belarus, Minsk, Belarus

[2]Institute of Biochemistry and Genetics, Ufa Research Centre, RAS, Ufa, Bashkortostan, Russia

[3]Estonian Biocentre, Tartu, Estonia

[4]Institute of Linguistics, Russian Academy of Sciences, Moscow, Russia

[5]School for Advanced Studies in the Humanities, Russian Presidential Academy of National Economy and Public Administration, Moscow, Russia

[6]Belarusian State University, Faculty of Applied Mathematics and Computer Science,

[7]Department of Probability Theory and Mathematical Statistics, Minsk, Belarus

Center of analytical and genetic engineering studies, Institute of Microbiology, National Academy of Sciences of Belarus, Minsk, Belarus

[8]Institute of Mathematical Statistics, University of Tartu, Tartu, Estonia

[9]Laboratory of Ethnogenomics, Institute of Molecular Biology, National Academy of Sciences of Armenia, Yerevan, 0014, Armenia

[10]Department of Evolutionary Biology, Institute of Molecular and Cell Biology, University of Tartu, Tartu, Estonia

[11]The Research Institute for Medical Genetics, 634050, Tomsk, Russia

[12]Department of Genetics and Fundamental Medicine of Bashkir State University, Ufa, Bashkortostan, Russia

[13]Institute of Fundamental Medicine and Biology, Kazan Federal University, Kazan, Russia

[14]Eco-Anthropologie et Ethnobiologie, UMR 7206 CNRS, MNHN, Université Paris Diderot, Sorbonne Universités, Muséum national d'Histoire naturelle, Musée de l'Homme, Paris, France

[15]Institute of Biological Problems of the North, Russian Academy of Sciences, Magadan, Russia

Supplementary Information Text (Linguistics)

Lipka Tatars

by Alexei Kassian

It is likely that Turkic-speaking Muslims, whose descendants are known as Lipka Tatars (or Belarusian-Polish-Lithuanian Tatars), begun to penetrate from the Golden Horde into the territory of the Grand Duchy of Lithuania since the beginning of the 13th century AD (Miškinienė 2005; Dybo 2006: 816) or even slightly earlier. As justified by historical sources (Sobczak 1984: 20), at the end of the 14th century, the Turkic-speaking Muslim migrants were already relatively numerous. Apparently, from this point forward, the Lipka Tatars were formed as a distinct ethnic group (Dumin 1991; Miškinienė 2005). The main waves of Turkic-speaking migrations in the Grand Duchy of Lithuania took place from the late 14th to the middle 16th centuries (Miškinienė 2005: 43–44).

The Lipka Tatars were gradually giving up their Turkic language, having shifted to the Slavic languages of surrounding communities: normally to Old Belarusian, sometimes to Polish. As follows from direct textual evidence, in the middle of the 16th century, a substantial part of Lipka Tatars has already abandoned their original language in favor of Belarusian and Polish (Dubiński 1972; Antonovich 1968: 10). It is probable that the original Turkic language (or languages?) was almost totally lost by the community already in the early 17th century (Miškinienė 2005).

There are known *ca*. 200 Lipka Tatar manuscripts in the Arabian script mostly of Muslim nature, dated back to the 17th–20th centuries (some holographs go back to the 16th century), (Jankowski 2003; Miškinienė 2005; Miškinienė 2012). The language of the Lipka

Tatar manuscripts is predominantly East Slavic, namely Old Belarusian; some texts are written down in Polish (frequently these are translated from Belarusian into Polish); see some editions: Antonovich 1968; Miškinienė 2001. Besides naturally determined Arabic, the manuscripts may contain passages in a Turkic language, for which see below.

Linguistic evidence for the original Turkic language of the Lipka Tatars may be revealed from Turkic words of the native cultural vocabulary, retained in the Slavic languages of the Lipka Tatars (such retentions are typical in the situation of a language shift unaccompanied by a full cultural shift), as well as from certain phonetic and grammatical traits of Lipka Tatars manuscripts.

As demonstrated by Dubiński (1972 with further references), the primary language of the main waves of Turkic-speaking migrations (14th–16th centuries) definitely belong to the Kipchak group of the Turkic language family. However, it is hard to propose a more exact position for the Lipka Tatar language within the aforementioned group; see Jankowski 2015 for similar conclusions in respect of Kipchak traits in Old Turkish passages of the Lipka Tatar manuscripts. It is also possible that the Turkic-speaking migrants actually spoke not a single, but several different, although closely related Kipchak lects: cf., e.g., the phonetic dissimilation *ll* > Lipka Tatar *ŋl* which is the same as in the Trakai Karaim language (Tenishev 2002: 283); on the other hand, Kipchak **y-* > Lipka Tatar *ǯ-* before *a, o* that is characteristic for modern Tatar, Karachay-Balkar, Kyrgyz and some other Kipchak lects, but not for Trakai Karaim where *y-* is retained (Tenishev 2002: 280).

The Kipchak group is one of the five main groups within the Turkic language family. The other traditionally distinguished groups are: Bulghar, Oghuz, Karluk, Siberian, plus some minor taxa; note that apparently the real genealogical classification is more complicated. The

Kipchak group consists of at least the following modern languages (the recently divergent lects are grouped):

- Karachay-Balkar, Kumyk,
- Karaim,
- Crimean Tatar (Middle "dialect"),
- Crimean Tatar (Steppe "dialect"),
- Tatar (several varieties), Bashkort,
- Nogai, Kyrgyz, Kazakh, Karakalpak.

See formal and informal classifications in Dybo 2013: 18; Hruschka et al. 2015; Tenishev 2002: 217; Johanson 1998: 82–83, which differ, however, in details. According to the lexicostatistical analysis by Dybo (2013: 18), the Proto-Kipchak language splits into the western (Karachay-Balkar, Kumyk, Karaim, Middle "dialect" of Crimean Tatar) and eastern (the rest of the lects) branches *ca*. the 9th century AD. Further detailed filiation of Kipchak lects cannot be proposed with certainty due to the following obstacles:

- dialects of individual languages are still poorly documented (particularly Swadesh wordlists are not collected);
- active contact-driven convergent processes within the Kipchak lects or between the Kipchak and other groups such as Oghuz and Karluk in the first half of the 2nd millennium AD that lead to linguistic homoplasy of various kinds (Tenishev 2002: 258–259; 736; Dybo 2006);
- some literary languages, which are much better described than their living dialects, are actually somewhat artificial being full of hidden loans (e.g., in the lexicostatistical tree in Dybo 2013: 18, the languages of the Karluk group,

namely Literary Uzbek and Literary Uygur, are included in the eastern Kipchak branch that seems historically unjustified).

Kipchak nature of the Lipka Tatar language is well confirmed by historical evidence. It is known that the main waves of the Lipka Tatars arrived from the Golden Horde and Post-Golden Horde khanates, such as the Crimean Khanate, in the 14th–16th centuries (see Pylypchuk 2014 with further references). Languages of the Golden Horde were predominantly Kipchak, including the official language of this state.

In the second half of the 2nd millennium, the Lipka Tatars were culturally and linguistically influenced by the Ottoman Empire, whose dominant languages were Turkish varieties, including the official language of the empire — Ottoman Turkish (Dubiński 1972: 86; Miškinienė 2005). Turkish belongs to the Oghuz group of the Turkic language family. Kipchak and Oghuz lects are not mutually intelligible, but nevertheless linguistically close enough to readily influence on each other, exchanging loanwords of various semantic fields (note that phonetic or grammatical loans between Kipchak and Oghuz are much more modest if exist at all).

It is very likely that the late and minor waves of Muslim migrants, having joined the Lipka Tatar community in the 16th–18th centuries, spoke a Turkized Kipchak language or simply Turkish (Miškinienė 2005: 43–44). Many Lipka Tatar manuscripts (17th–20th centuries) contain Turkic passages whose language is identified as (Old) Turkish, see, e.g., Antonovich 1968: 17–22; Miškinienė 2008; Miškinienė & Durgut 2009; Jankowski 2015. Discussed Turkish passages are the result of intensive communications with the Ottoman Empire and should not be regarded as an evidence for Oghuz nature of the original Lipka Tatar language.

It is interesting that there exists a small number of Lipka Tatar manuscripts with Turkic portions other than (Old) Turkish, namely in an archaic language of the Karluk group: the language of these texts is called “Chagatai” by Szynkiewicz (1935: 141; followed by Kryczyński 1938), “East Turkic” by Jankowski (2015), “Khorezmian and Chagatai” by Tenishev (1997), and “Old Uygur” by Zajączkowski (1951: 311). Additionally it is stated by Jankowski (2003: 114; 2015) that Turkish portions themselves may contain not only borrowed Kipchak linguistic element, but also Chagatai ones. The Karluk group of the Turkic family consists of modern closely related Uzbek and Uygur (both with dialect varieties); the archaic stage of the Karluk group is attested as the extinct literary languages: Khorezmian (13th–14th centuries AD) and Chagatai (15th–16th centuries AD), Tenishev 1996; Johanson 1998: 86. The Karluk group is apparently distinct from the Kipchak one, although Karluk lects having been heavily Kipchakized during their known history.

The question of archaic Karluk (which could be labeled “Chagatai” for the sake of convenience) elements in the Lipka Tatar manuscripts requires additional investigations. The revealed Chagatai traces can be explained in two ways. (1) In the mid 2nd millennium, Kipchak-speaking Lipka Tatars borrowed the writing religious tradition from the Crimean Khanate where Chagatai was one of the official languages, but latter shifted to the Turkish-speaking Ottoman tradition. (2) Some part of the Lipka Tatars was originally Karluk-speaking, Chagatai portions in the manuscripts are thus remnants of their language and culture. The first solution is definitely more natural.

Summing up, at least the majority of the Lipka Tatar ancestors is supposed to be Kipchak-speaking.

References (Linguistics)

Antonovich, A.K. 1968. *Belorusskie teksty, pisannye arabskim pis'mom, i ikh grafiko-orfograficheskaya sistema*. Vilnius: VGU.

Dubiński, A. 1972. Zametki o yazyke litovskikh tatar. *Voprosy yazykoznaniya* 1: 82–88.

Dumin, S.V. 1991. Tatarskie knyaz'ya v Velikom knyazhestve Litovskom. *Acta Baltico-Slavica* 20: 7–49.

Dybo, Anna V. 2006. Khronologiya tyurkskikh yazykov i lingvisticheskie kontakty rannikh tyurkov. In: E.R. Tenishev, A.V. Dybo (eds.). *Sravnitel'no-istoricheskaya grammatika tyurkskikh yazykov*. *Pratyurkskij yazyk-osnova*. *Kartina mira pratyurkskogo etnosa po dannym yazyka*. Moscow: Nauka: 766–817.

Dybo, Anna V. 2013. *Etimologicheskij slovar' tyurkskikh yazykov*. Vol. 9: *Etimologicheskij slovar' bazisnoj leksiki tyurkskikh yazykov*. Astana: Russian Academy of Sciences.

Hruschka, Daniel J., Simon Branford, Eric D. Smith, Jon Wilkins, Andrew Meade, Mark Pagel, Tanmoy Bhattacharya. 2015. Detecting regular sound changes in linguistics as events of concerted evolution. *Current Biology* 25: 1–9.

Jankowski, Henryk. 2003. Polish-Lithuanian-Belarusian Tatar documents. *Materialia Turcica* 24: 113–144.

Jankowski, Henryk. 2015. Cechy graficzne i językowe tekstów turkijskich w zapisie kopistów polsko-tatarskich [Grapic and linguistic features of Turkic texts as recorded by Polish-Tatar copyists]. In: Joanna Kulwicka-Kamińska & Czesław Łapicz (eds.). *Tefsir Tatarów Wielkiego Księstwa Litewskiego: Teoria i praktyka badawcza* [*The Tafsir of the Tatars of the Grand Duchy of Lithuania: Theory and Research*]. Toruń: Wydział Filologiczny UMK: 139–171.

Johanson, Lars. 1998. The history of Turkic. In: Lars Johanson, Éva Á. Csató (eds.). *The Turkic languages*. London / New York: Routledge: 81–125.

Kryczyński, Stanisław. 1938. *Tatarzy litewscy: próba monografii historyczno-etnograficznej* (= *Rocznik Tatarski* 3). Warszawa: Wydanie Rady Centralnej Związku Kulturalno-Oświatowego Tatarów Rzeczypospolitej Polskiej.

Miškinienė, Galina. 2001. *Drevnejshie rukopisi litovskikh tatar (Grafika. Transliteratsiya. Perevod. Struktura i soderzhanie tekstov).* Vilnius: VGU.

Miškinienė, Galina. 2005. Ocherk istorii i kul'tury litovskikh tatar [A Study of the History and Culture of Lithuanian Tatars]. *Diaspory* 2: 40–61.

Miškinienė, Galina. 2008. Turetsko-pol'skij slovarik iz kitaba Yakuba Khasenevicha (1840 g.). In: *Orientas Lietuvos Didžiosios Kunigaikštijos visuomenės tradicijoje: totoriai ir karaimai*. Vilnius: Vilniaus universiteto leidykla: 105–121.

Miškinienė, Galina. 2012. Svodnyj katalog arabskoalfavitnykh rukopisej litovskikh tatar: L'vovskaya nauchnaya biblioteka im. V. Stefanika NAN Ukrainy. *Naujausi kalbų ir kultūrų tyrimai*. Vilnius: Europos kalbų ir kultūrų dialogo tyrėjų asociacija: 45–58.

Miškinienė, Galina, Huseyin Durgut. 2009. Legenda "Miradzh" iz kitaba Ivana Lutskevicha. *Bibliotheca Archivi Lithuanici* 7: *Lietuvos Didžiosios Kunigaikštystės kalbos, kultūros ir raštijos tradicijos*. Vilnius: Lietuvių kalbos institutas: 357–375.

Pylypchuk, Yaroslav. 2014. Tatary v Velikom Knyazhestve Litovskom (pravoslavnye i musul'mane): assimilyatsiya i adaptatsiya. *Srednevekovye tyurko-tatarskie gosudarstva* 6. Kazan: 101-110.

Sobczak, Jacek. 1984. *Położenie prawnie ludności tatarskiej w Wielkim Księstwie Litewskim*. Warszawa / Poznań: Państwowe wydawnictwo naukowe.

Szynkiewicz, Jakób. 1935. Literatura religijna Tatarów litewskich i jej pochodzenie. *Rocznik Tatarski* 2: 138–144.

Tenishev, E. R. (ed.). 1996. *Yazyki mira: Tyurkskie yazyki* [*Languages of the Word: Turkic Languages*]. Bishkek: Kyrgyztan.

Tenishev, E. R. 1997. Razgovornyj yazyk pol’sko-belorusskikh tatar XV–XVI vv. *Izvestiya AN: Seriya literatury i yazyka* 56(6): 3–8.

Tenishev, E. R. (ed.). 2002. *Sravnitel’no-istoricheskaya grammatika tyurkskikh yazykov. Regional’nye rekonstruktsii*. Moscow: Nauka.

Zajączkowski, Ananjasz. 1951. Tak zwany chamaił tatarski ze zbiorów rękopisów w Warszawie. *Sprawozdania z Czynności i Posiedzeń PAU* 52(4): 307–313.

Supplementary Information Text (Genetics)

Summary of the studies published in Pankratov, V., Kushniarevich, A., Chebotarev, L., Metspalu, E. & Davydenko, O. Formirovanie pula mitokhondrialnoi DNK Belorusskih Tatar: dalnie migracii i smeshenie genofondov [Mitochondrial DNA gene pool shaping of Belarusian Tatars: long-distance migrations and admixture]. Dokl. Nac. Akad. Nauk 58, 82–87 (2014) and in Pankratov, V., Kushniarevich, A. & Davydenko, O. Polimorfizm markerov Y khromosomy v populiacii Belorusskih Tatar [Genetic polymorphism of Y-chromosomal markers in the population of Belarusian Tatars]. Dokl. Akad. Nauk Belarusi **58,** 94–100 (2014).

The aim of the first study was to characterize mtDNA diversity in the ethno-religious group – Belarusian Lipka Tatars (BLT), who reside currently in the Belarus territory. Altogether 91 buccal swab samples from unrelated individuals who identified themselves as Belarusian Lipkas were collected initially, 80 of these were selected for further analyses based on questionnaire information and detailed investigation of volunteers genealogies. Collection of the material was performed after the informed consent was obtained from each participant. DNA samples were extracted using proteinase K and phenol-chloroform method. Sequencing of HVSI along with genotyping of informative positions in HVSII and coding region of mtNDA was done. Substitutions were scored against the Reconstructed Sapiens Reference Sequence (RSRS)[1]. Haplogroups were defined according to the human mtDNA phylogeny (phylotree.org mtDNA tree Build 16 (19 Feb 2014)[2].

Phylogeny of mtDNA haplogroups and their frequencies in BLT are given in Figure 1. Altogether, 33 haplogroups were detected in the studied sample. MtDNA haplpogroups can be conditionally divided into East Eurasian and West Eurasian according to their geographic distribution[3].

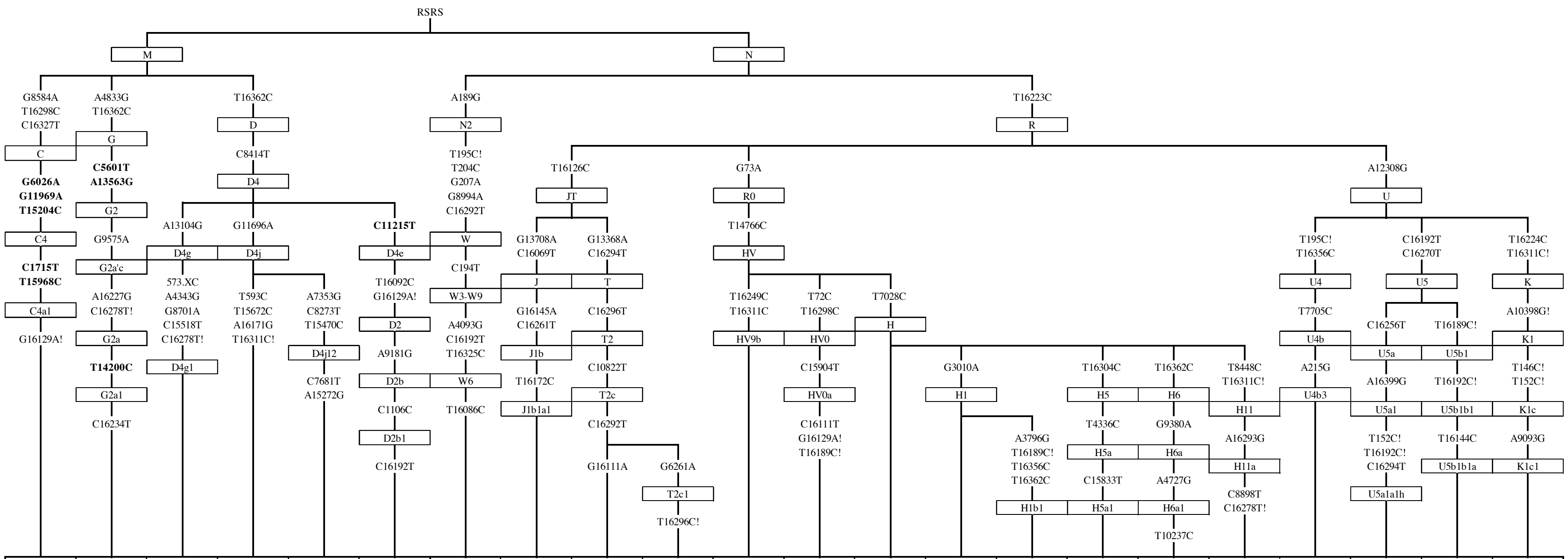

	C4a1	G2a1	D4g1	D4j*	D4j12	D2b1	W6	J1b1a1	T2c*	T2c1	HV9b	HV0a	H*	H1*	H1b1	H5a1	H6a1a4	H11a1	U4b3	U5a1a1h	U5b1b1a	K1c1
Haplotypes, total N	4	11	1	1	6	3	4	1	2	1	1	3	10	3	1	10	7	6	1	1	2	1
%	5	13,8	1,3	1,3	7,5	3,8	5	1,3	2,5	1,3	1,3	3,8	12,5	3,8	1,3	12,5	8,8	7,5	1,3	1,3	2,5	1,3
Unique haplotypes, N	3	3	1	1	2	1	1	1	2	1	1	1	5	2	1	2	1	2	1	1	1	1
Matching haplotypes in Belarusians, N	0	0	0	0	0	0	0	2	0	0	0	0	21[a]	11[a]	1	0	1	2	2	0	2	0
%	0	0	0	0	0	0	0	0,7	0	0	0	0	7,9	4,1	0,4	0	0,4	0,7	0,7	0	0,7	0
	East Eurasian haplogroups						West Eurasian haplogroups															

Figure 1. MtDNA phylogeny of Belarusian Lipka Tatars. Mutations are scored against the RSRS and indicated on the branches: in usual font are positions that were genotyped in all samples of a respective haplogroup; in bold are positions that are known only for samples that were sequenced completely; "!" indicates back mutations. Frequencies of mtDNA haplogroups in Belarusians (N=267) are also given. [a] – samples that belong to different sub variants of haplogroup H with HVSI motifs matching to those of Belarusian Lipka Tatars.

First group includes variants of the macro haplogroup M: C4, G2, D4 and D2 – altogether 11 different haplotypes – and makes 1/3 of the mtDNA pool of BLT (Figure 1). The most frequent East Eurasian haplogroups in BLT are G2a1 and D4j. Those haplogroups are spread nowadays in Central and East Asia[4] and virtually absent from East Europe[5,6].

The second group unites sub-variants of the N macro haplogroup: W, J, T, HV, U and K (24 haplotypes altogether). Among the most common West Eurasian mtDNA haplogroups found in BLT are H5a1, H6a1a4 and H11a1. Those haplogroups are widely spread among Europeans although at low frequency[6,7].

Phylogenetic analysis of East Eurasian haplogroups found in BLT and Belarusians revealed their distinctive origin and did not support matrilineal gene flow from BLT to Belarusians marked by those lineages. On the other hand, mtDNAs of West Eurasian origin indicate likely matrilineal admixture between the two populations or between ancestors of BLT and West Eurasians.

Thus, one-third of the maternal gene pool of Belarusian Lipka Tatars can be classified as East Eurasian – a feature that differ them from surrounding populations like Belarusians, Poles, Lithuanians, and indicate an admixed nature of their gene pool; moderate matrilineal gene flow between Belarusians and BLT; a signs of founder effect and genetic drift as seen in a number of identical tmDNA haplotypes.

The aim of the second study was to characterize Y-chromosome variation in Belarusian Lipka Tatars with respect to surrounding host-population.

74 unrelated BLT men were genotyped for 26 Y-chromosomal biallelic markers: M89, M201, P15, M170, M223, P37.2, 12f2, M267, P58, M172, M410, M67, M12, M9, M231, Tat, 92R7, M242, M25, M346, M378, SRY1532.2, M458, M269, M412 and M478 following the

hierarchy of haplogroups in the Y-chromosome phylogeny (http://www.phylotree.org/Y/tree/) (van Oven et al., 2014). In addition, 17 Y-STR markers from the AmpFLSTR® Yfiler® kit (Applied Biosystems) were determined.

The haplogroup composition and their frequency in BLT sample are given in Table 1. For the purpose of comparison frequencies of respective haplogroups in Belarusians are also given.

Table 1. Frequencies of different Y-chromosome haplogroups among Belarusian Lipka Tatars.

Y-chromosome haplogroup	Number of individuals	Percentage	Number of distinct Y-STR haplotypes	Percentage among Belarusians[5]
G2a-P15	3	4.1	2	1.4
I2a-M223	1	1.4	1	1.2
I2a-P37.2	0	0.0	0	17.2
J1a-P58	4	5.4	2	1.2*
J2a(xM67)	12	16.2	8	2.7*
J2a-M67	1	1.4	1	
J2b-M12	2	2.7	2	
N-Tat	2	2.7	2	9.6
Q1a-M25	1	1.4	1	0.2*
Q1a-M346	3	4.1	3	
Q1b-M378	2	2.7	1	
R1a(xM458)	20	27.0	12	31.6
R1a-M458	16	21.6	11	14.5
R1b-M478	2	2.7	2	0.4
R1b-M412	5	6.8	2	5.7*
Total	74		50	

* – frequencies of haplogroups G2a-P15, J1-M267, J2-M172, Q-M242, R1a(xM458) and R1b-M269 respectively as these individuals were not genotyped for deeper markers.

Majority of the Y-chromosome diversity in BLT is covered by R1a(xM458), R1a-M458, J2a(xM67) and sub-variants of Q haplogroups (Table 1). The most prominent differences between patrilineal gene pool of BLT and host population are higher share of subclades of J2 and Q as well as lower percentage of N-Tat and absence of I2a-P37.2 in BLT.

Analysis of Y-STR variation of R1a(M458) and R1a(M558) haplogroups revealed number of shared haplotypes between BLT and Belarusians that might indicate patrilineal gene flow between the two populations marked by those lineages.

To compare the overall Y-chromosome composition of the BLT to that of other populations we applied principal component analysis (PCA) based on Y-chromosome haplogroup frequencies in East Europeans, Volga-Uralic region, Caucasus and Central Asia, Mongolia. Resulted PCA plot is shown in Figure 1.

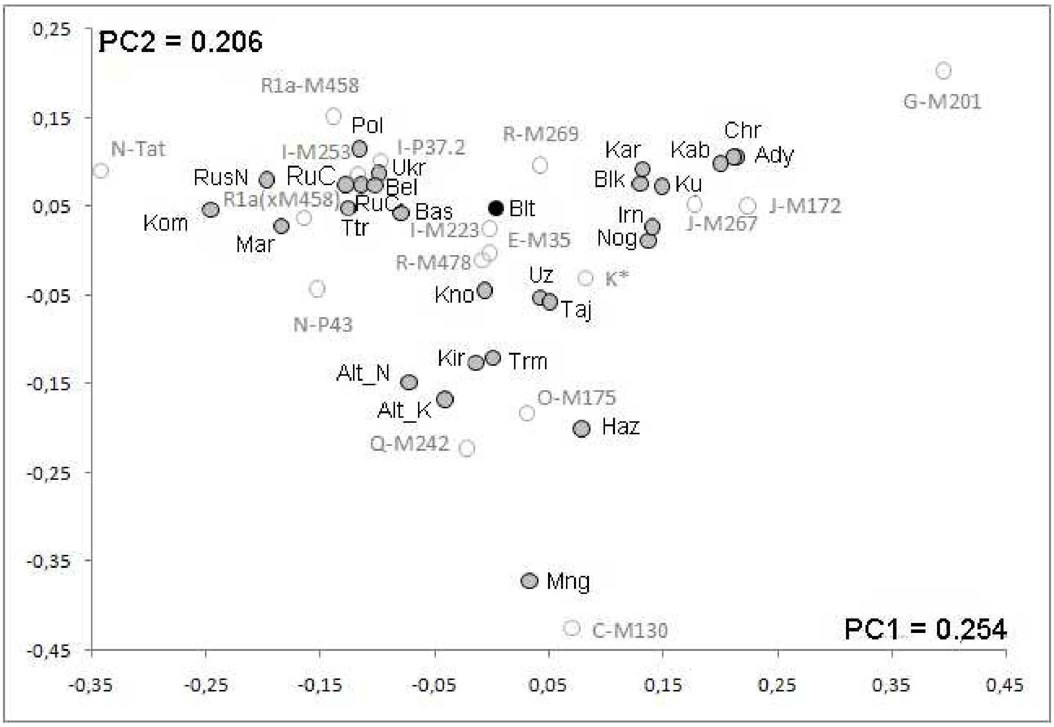

Figure 1. PCA based on Y-chromosome haplogroup frequencies[5,8–14]. Populations are abbreviated as in Supplementary Table 4, except RusN – Russians from the northern part of the European region of Russia and Alt_N – northern Altaians (Chelkan, Kumandin and Tubalar populations pulled together). Y-chromosome haplogroups are shown in grey color.

As can be seen in Figure 1 BLT occupy an intermediate position between population from Eastern Europe and the Caucasus along the PC1 axis due to a relatively high share of

haplogroup R1a in former and haplogroups J-M172, J-M267 and G201 in latter. The PC2 axis of the plot can be considered as approximation of the measure of the proportion of East Eurasian component in the gene pools of populations; BLT take a position which is close to that of Volga Tatars, Bashkirs and Komis. Taken together, the PCA results indicated an admixed origin of patrilineal pool of BLT as it harbors haplogroups, which are typical for Caucasus, Central Asia and Siberia along with those common currently in Europeans.

Altogether, our exploratory studies revealed that Belarusian Lipka Tatars bear substantial share of East Eurasian ancesptry in their paternal and maternal gene pools – a feature that differ Belarusian Lipkas from their current neighboring populations.

References (Genetics)

1. Behar, D. M. *et al.* A 'Copernican' reassessment of the human mitochondrial DNA tree from its root. *Am. J. Hum. Genet.* **90,** 675–684 (2012).
2. van Oven, M. & Kayser, M. Updated comprehensive phylogenetic tree of global human mitochondrial DNA variation. *Hum. Mutat.* **30,** E386–E394 (2009).
3. Underhill, P. A. & Kivisild, T. Use of y chromosome and mitochondrial DNA population structure in tracing human migrations. *Annu. Rev. Genet.* **41,** 539–564 (2007).
4. Derenko, M. *et al.* Origin and post-glacial dispersal of mitochondrial DNA haplogroups C and D in northern Asia. *PloS One* **5,** e15214 (2010).
5. Kushniarevich, A. *et al.* Uniparental genetic heritage of belarusians: encounter of rare middle eastern matrilineages with a central European mitochondrial DNA pool. *PloS One* **8,** e66499 (2013).
6. Mielnik-Sikorska, M. *et al.* The history of Slavs inferred from complete mitochondrial genome sequences. *PloS One* **8,** e54360 (2013).
7. Malyarchuk, B. *et al.* The peopling of Europe from the mitochondrial haplogroup U5 perspective. *PloS One* **5,** e10285 (2010).
8. Balanovsky, O. *et al.* Two sources of the Russian patrilineal heritage in their Eurasian context. *Am. J. Hum. Genet.* **82,** 236–250 (2008).
9. Rebała, K. *et al.* Y-STR variation among Slavs: evidence for the Slavic homeland in the middle Dnieper basin. *J. Hum. Genet.* **52,** 406–414 (2007).
10. Rootsi, S. *et al.* Phylogeography of Y-chromosome haplogroup I reveals distinct domains of prehistoric gene flow in europe. *Am. J. Hum. Genet.* **75,** 128–137 (2004).
11. Tambets, K. *et al.* The western and eastern roots of the Saami--the story of genetic 'outliers' told by mitochondrial DNA and Y chromosomes. *Am. J. Hum. Genet.* **74,** 661–682 (2004).

12. Dulik, M. C. *et al*. Mitochondrial DNA and Y chromosome variation provides evidence for a recent common ancestry between Native Americans and Indigenous Altaians. *Am. J. Hum. Genet.* **90,** 229–246 (2012).
13. Di Cristofaro, J. *et al*. Afghan Hindu Kush: where Eurasian sub-continent gene flows converge. *PloS One* **8,** e76748 (2013).
14. Yunusbayev, B. *et al*. The Caucasus as an asymmetric semipermeable barrier to ancient human migrations. *Mol. Biol. Evol.* **29,** 359–365 (2012).

Supplementary Information Text (Material and Methods)

In the whole genome SNP variation analyses we have used six samples of Belarusian Lipka Tatars (BLT) genotyped on Illumina HumanOmniExpress-24 v1.0 BeadChip that includes around 730 thousand SNPs. Although this sample size is small, we believe that conclusions drawn in this study are reliable and fairly applicable to the BLT population.

Dataset

BLT samples genotyped genome-wide were selected from different regions of Belarus so to increase the diversity of analyzed dataset. According to self-reported information as well as to our analysis for cryptic kinship, those samples are not relatives in at least three generations.

Genetic homogeneity of the BLT dataset

Analyses based on genotypes' data as well as haplotype-based approaches indicate the homogeneity of BLT dataset used in this study.

a) PC plot PC1vsPC2 shown in Figure 2A in the main text reveals that BLT do not overlap, yet nevertheless form a tight cluster of their own;

b) ADMIXTURE plot (k6) (Figure 2B in the main text) shows that all six individuals bear similar ancestral proportions to each other – another indication of homogeneity of their autosomal genomes and the lack of recent admixture with equally highly homogeneous, though different, Belarusians.

c) fineSTRUCTURE dendrogram (Supplementary Fig. 12) is based on information on genomic chunks "copied" by a recipient population from a range of other

populations (donors). According to the dendrogram, BLTs are differentiated from other populations and form a single cluster.

Hence, analyses performed in this study suggest that though the sample set of six BLT is limited, it is homogeneous and representative for Lipkas.

Supplementary Information Text (Full List of References for Figure 1)

1. Rootsi, S. *et al.* A counter-clockwise northern route of the Y-chromosome haplogroup N from Southeast Asia towards Europe. *Eur. J. Hum. Genet. EJHG* **15,** 204–211 (2007).
2. Balaresque, P. *et al.* Y-chromosome descent clusters and male differential reproductive success: young lineage expansions dominate Asian pastoral nomadic populations. *Eur. J. Hum. Genet.* **23,** 1413–1422 (2015).
3. Heyer, E. *et al.* Patrilineal populations show more male transmission of reproductive success than cognatic populations in Central Asia, which reduces their genetic diversity. *Am. J. Phys. Anthropol.* **157,** 537–543 (2015).
4. Kushniarevich, A. *et al.* Uniparental genetic heritage of Belarusians: encounter of rare Middle Eastern matrilineages with a Central European mitochondrial DNA pool. *PloS One* **8,** e66499 (2013).
5. Kushniarevich, A. *et al.* Genetic heritage of the Balto-Slavic speaking populations: A synthesis of autosomal, mitochondrial and Y-chromosomal data. *PLoS ONE* **10,** e0135820 (2015).
6. Pankratov, V., Kushniarevich, A. & Davydenko, O. Polimorfizm markerov Y khromosomy v populiacii Belorusskih Tatar. *Dokl. Akad. Nauk Belarusi* **58,** 94–100 (2014) (in Russian).
7. Lappalainen, T. *et al.* Migration waves to the Baltic Sea region. *Ann. Hum. Genet.* **72,** 337–348 (2008).
8. Lappalainen, T. *et al.* Population structure in contemporary Sweden--a Y-chromosomal and mitochondrial DNA analysis. *Ann. Hum. Genet.* **73,** 61–73 (2009).
9. Zerjal, T. *et al.* Geographical, linguistic, and cultural influences on genetic diversity: Y-chromosomal distribution in Northern European populations. *Mol. Biol. Evol.* **18,** 1077–1087 (2001).
10. Zerjal, T. *et al.* The genetic legacy of the Mongols. *Am. J. Hum. Genet.* **72,** 717–721 (2003).
11. Malyarchuk, B. *et al.* Ancient links between Siberians and Native Americans revealed by subtyping the Y chromosome haplogroup Q1a. *J. Hum. Genet.* **56,** 583–588 (2011).
12. Dulik, M. C., Osipova, L. P. & Schurr, T. G. Y-chromosome variation in Altaian Kazakhs reveals a common paternal gene pool for Kazakhs and the influence of Mongolian expansions. *PloS One* **6,** e17548 (2011).

13. Di Cristofaro, J. *et al*. Afghan Hindu Kush: where Eurasian sub-continent gene flows converge. *PloS One* **8,** e76748 (2013).
14. Underhill, P. A. *et al*. Separating the post-Glacial coancestry of European and Asian Y chromosomes within haplogroup R1a. *Eur. J. Hum. Genet. EJHG* **18,** 479–484 (2010).
15. Karmin, M. *et al*. A recent bottleneck of Y chromosome diversity coincides with a global change in culture. *Genome Res*. (2015). doi:10.1101/gr.186684.114
16. Underhill, P. A. *et al*. The phylogenetic and geographic structure of Y-chromosome haplogroup R1a. *Eur. J. Hum. Genet. EJHG* (2014). doi:10.1038/ejhg.2014.50
17. Mielnik-Sikorska, M. *et al*. The history of Slavs inferred from complete mitochondrial genome sequences. *PloS One* **8,** e54360 (2013).
18. Rebała, K. *et al*. Y-STR variation among Slavs: evidence for the Slavic homeland in the middle Dnieper basin. *J. Hum. Genet*. **52,** 406–414 (2007).
19. Balanovsky, O. *et al*. Parallel evolution of genes and languages in the Caucasus region. *Mol. Biol. Evol*. **28,** 2905–2920 (2011).
20. Myres, N. M. *et al*. A major Y-chromosome haplogroup R1b Holocene era founder effect in Central and Western Europe. *Eur. J. Hum. Genet. EJHG* **19,** 95–101 (2011).
21. Rootsi, S. *et al*. Distinguishing the co-ancestries of haplogroup G Y-chromosomes in the populations of Europe and the Caucasus. *Eur. J. Hum. Genet. EJHG* **20,** 1275–1282 (2012).
22. Semino, O. *et al*. Origin, diffusion, and differentiation of Y-chromosome haplogroups E and J: inferences on the Neolithization of Europe and later migratory events in the Mediterranean area. *Am. J. Hum. Genet*. **74,** 1023–1034 (2004).
23. Chaubey, G. *et al*. Genetic affinities of the Jewish populations of India. *Sci. Rep*. **6,** 19166 (2016).
24. Rootsi, S. *et al*. Phylogeography of Y-chromosome haplogroup I reveals distinct domains of prehistoric gene flow in europe. *Am. J. Hum. Genet*. **75,** 128–137 (2004).
25. Peričić, M. *et al*. High-resolution phylogenetic analysis of southeastern Europe traces major episodes of paternal gene flow among Slavic populations. *Mol. Biol. Evol*. **22,** 1964–1975 (2005).
26. Battaglia, V. *et al*. Y-chromosomal evidence of the cultural diffusion of agriculture in Southeast Europe. *Eur. J. Hum. Genet. EJHG* **17,** 820–830 (2009).
27. Marjanovic, D. *et al*. The peopling of modern Bosnia-Herzegovina: Y-chromosome haplogroups in the three main ethnic groups. *Ann. Hum. Genet*. **69,** 757–763 (2005).

28. Al-Zahery, N. *et al.* In search of the genetic footprints of Sumerians: a survey of Y-chromosome and mtDNA variation in the Marsh Arabs of Iraq. *BMC Evol. Biol.* **11,** 288 (2011).
29. Chiaroni, J. *et al.* The emergence of Y-chromosome haplogroup J1e among Arabic-speaking populations. *Eur. J. Hum. Genet.* **18,** 348–353 (2010).
30. Balanovsky, O. *et al.* Two sources of the Russian patrilineal heritage in their Eurasian context. *Am. J. Hum. Genet.* **82,** 236–250 (2008).
31. Luca, F. *et al.* Y-chromosomal variation in the Czech Republic. *Am. J. Phys. Anthropol.* **132,** 132–139 (2007).
32. Mirabal, S. *et al.* Y-chromosome distribution within the geo-linguistic landscape of northwestern Russia. *Eur. J. Hum. Genet. EJHG* **17,** 1260–1273 (2009).
33. Semino, O. *et al.* The genetic legacy of Paleolithic Homo sapiens sapiens in extant Europeans: a Y chromosome perspective. *Science* **290,** 1155–1159 (2000).
34. Tambets, K. *et al.* The western and eastern roots of the Saami--the story of genetic 'outliers' told by mitochondrial DNA and Y chromosomes. *Am. J. Hum. Genet.* **74,** 661–682 (2004).
35. Rebała, K. *et al.* Forensic analysis of polymorphism and regional stratification of Y-chromosomal microsatellites in Belarus. *Forensic Sci. Int. Genet.* **5,** e17-20 (2011).
36. Bermisheva, M., Tambets, K., Villems, R. & Khusnutdinova, E. [Diversity of mitochondrial DNA haplotypes in ethnic populations of the Volga-Ural region of Russia]. *Mol. Biol. (Mosk.)* **36,** 990–1001 (2002).
37. Grzybowski, T. *et al.* Complex interactions of the Eastern and Western Slavic populations with other European groups as revealed by mitochondrial DNA analysis. *Forensic Sci. Int. Genet.* **1,** 141–147 (2007).
38. Karachanak, S. *et al.* Bulgarians vs the other European populations: a mitochondrial DNA perspective. *Int. J. Legal Med.* **126,** 497–503 (2012).
39. Kasperaviciūte, D., Kucinskas, V. & Stoneking, M. Y chromosome and mitochondrial DNA variation in Lithuanians. *Ann. Hum. Genet.* **68,** 438–452 (2004).
40. Malyarchuk, B. A. *et al.* Mitochondrial DNA variability in Poles and Russians. *Ann. Hum. Genet.* **66,** 261–283 (2002).
41. Malyarchuk, B. A. *et al.* Mitochondrial DNA variability in Bosnians and Slovenians. *Ann. Hum. Genet.* **67,** 412–425 (2003).
42. Malyarchuk, B. *et al.* Mitochondrial DNA Phylogeny in Eastern and Western Slavs. *Mol. Biol. Evol.* **25,** 1651–1658 (2008).

43. Pliss, L. *et al*. Mitochondrial DNA portrait of Latvians: towards the understanding of the genetic structure of Baltic-speaking populations. *Ann. Hum. Genet.* **70,** 439–458 (2006).
44. Pala, M. *et al*. Mitochondrial DNA Signals of Late Glacial Recolonization of Europe from Near Eastern Refugia. *Am. J. Hum. Genet.* **90,** 915–924 (2012).
45. Fernandes, V. *et al*. The Arabian Cradle: Mitochondrial Relicts of the First Steps along the Southern Route out of Africa. *Am. J. Hum. Genet.* **90,** 347–355 (2012).
46. Fanti, S. D. *et al*. Fine Dissection of Human Mitochondrial DNA Haplogroup HV Lineages Reveals Paleolithic Signatures from European Glacial Refugia. *PLOS ONE* **10,** e0144391 (2015).
47. Olivieri, A. *et al*. Mitogenomes from Two Uncommon Haplogroups Mark Late Glacial/Postglacial Expansions from the Near East and Neolithic Dispersals within Europe. *PLOS ONE* **8,** e70492 (2013).
48. Roostalu, U. *et al*. Origin and expansion of haplogroup H, the dominant human mitochondrial DNA lineage in West Eurasia: the Near Eastern and Caucasian perspective. *Mol. Biol. Evol.* **24,** 436–448 (2007).
49. Yunusbayev, B. *et al*. The Caucasus as an asymmetric semipermeable barrier to ancient human migrations. *Mol. Biol. Evol.* **29,** 359–365 (2012).
50. Malyarchuk, B., Derenko, M., Denisova, G. & Kravtsova, O. Mitogenomic diversity in Tatars from the Volga-Ural region of Russia. *Mol. Biol. Evol.* **27,** 2220–2226 (2010).
51. Malyarchuk, B. A., Perkova, M. A. & Derenko, M. V. On the origin of Mongoloid component in the mitochondrial gene pool of Slavs. *Russ. J. Genet.* **44,** 344–349 (2008).
52. Derenko, M. *et al*. Complete mitochondrial DNA analysis of eastern Eurasian haplogroups rarely found in populations of northern Asia and eastern Europe. *PloS One* **7,** e32179 (2012).
53. Derenko, M. *et al*. Y-chromosome haplogroup N dispersals from south Siberia to Europe. *J. Hum. Genet.* **52,** 763–770 (2007).
54. Irwin, J. A. *et al*. The mtDNA composition of Uzbekistan: a microcosm of Central Asian patterns. *Int. J. Legal Med.* **124,** 195–204 (2010).
55. Pshenichnov, A. *et al*. Genetic affinities of Ukrainians from the maternal perspective. *Am. J. Phys. Anthropol.* **152,** 543–550 (2013).
56. Nikitin, A. G., Newton, J. R. & Potekhina, I. D. Mitochondrial haplogroup C in ancient mitochondrial DNA from Ukraine extends the presence of East Eurasian genetic lineages in Neolithic Central and Eastern Europe. *J. Hum. Genet.* **57,** 610–612 (2012).

57. Malyarchuk, B. *et al*. The peopling of Europe from the mitochondrial haplogroup U5 perspective. *PloS One* **5,** e10285 (2010).
58. Yao, Y.-G., Kong, Q.-P., Wang, C.-Y., Zhu, C.-L. & Zhang, Y.-P. Different matrilineal contributions to genetic structure of ethnic groups in the Silk Road region in China. *Mol. Biol. Evol.* **21,** 2265–2280 (2004).
59. Derenko, M. *et al*. Western Eurasian ancestry in modern Siberians based on mitogenomic data. *BMC Evol. Biol.* **14,** 217 (2014).
60. Starikovskaya, E. B. *et al*. Mitochondrial DNA diversity in indigenous populations of the southern extent of Siberia, and the origins of native American haplogroups. *Ann. Hum. Genet.* **69,** 67–89 (2005).
61. Derenko, M. V. *et al*. Diversity of mitochondrial DNA lineages in South Siberia. *Ann. Hum. Genet.* **67,** 391–411 (2003).
62. Gokcumen, O. *et al*. Genetic variation in the enigmatic Altaian Kazakhs of South-Central Russia: insights into Turkic population history. *Am. J. Phys. Anthropol.* **136,** 278–293 (2008).
63. Sato, T., Razhev, D., Amano, T. & Masuda, R. Genetic features of ancient West Siberian people of the Middle Ages, revealed by mitochondrial DNA haplogroup analysis. *J. Hum. Genet.* **56,** 602–608 (2011).
64. Губина, М. А., Дамба, Л. Д., Бабенко, В. Н., Ромащенко, А. Г. & Воевода, М. И. Гаплотипическое разнообразие мтднк и Y-хромосомы в популяциях Алтае-Саянского региона. *Генетика* **49,** 376–391 (2013).
65. Sukernik, R. I. *et al*. Mitochondrial genome diversity in the Tubalar, Even, and Ulchi: contribution to prehistory of native Siberians and their affinities to Native Americans. *Am. J. Phys. Anthropol.* **148,** 123–138 (2012).
66. Fedorova, S. A. *et al*. Autosomal and uniparental portraits of the native populations of Sakha (Yakutia): implications for the peopling of Northeast Eurasia. *BMC Evol. Biol.* **13,** 127 (2013).
67. Kong, Q.-P. *et al*. Mitochondrial DNA sequence polymorphisms of five ethnic populations from northern China. *Hum. Genet.* **113,** 391–405 (2003).
68. Lee, H. Y. *et al*. East Asian mtDNA haplogroup determination in Koreans: haplogroup-level coding region SNP analysis and subhaplogroup-level control region sequence analysis. *Electrophoresis* **27,** 4408–4418 (2006).

Supplementary Information Figures Legends

Supplementary Figure 1. Phylogeny and frequency of Y-chromosome haplogroups in Belarusian Lipka Tatars. In red are shown biallelic markers genotyped in this study.

Supplementary Figure 2. Y-STR Median Networks for haplogroup G2a-U1 in Belarusian Lipka Tatars and other Eurasian populations. The network was constructed based on 10-loci haplotypes. Haplotypes differing from BLT haplotypes by no more than 3 mutation steps were included. The complete network that includes all haplotypes given in Supplementary Table 4, is shown in the bottom right panel. In both cases markers were given the following weights: DYS19 – 3, DYS385a – 3, DYS385b – 3, DYS389I – 4, DYS389II – 2, DYS390 – 3, DYS391 – 4, DYS392 – 5, DYS393 – 5, DYS439 – 3. Extended information related to the networks (population names, color codes, number of samples and Y-STRs used) can be found in Supplementary Table 4.

Supplementary Figure 3. Y-STR Median Networks for haplogroup J1-P58 in Belarusian Lipka Tatars and other Eurasian populations. The network was constructed based on 10-loci haplotypes. All haplotypes given in Supplementary Table 4 are included. Markers were given the following weights: DYS19 – 5, DYS385a – 2, DYS385b –1, DYS389I – 4, DYS389II – 2, DYS390 – 4, DYS391 – 3, DYS392 – 5, DYS393 – 5, DYS439 – 2. Extended information related to the networks (population names, color codes, number of samples and Y-STRs used) can be found in Supplementary Table 4.

Supplementary Figure 4. Y-STR Median Networks for haplogroup J2a(xM67) in Belarusian Lipka Tatars and other Eurasian populations. The network was constructed based on 10-loci haplotypes. Haplotypes differing from BLT haplotypes by no more than 3 mutation steps were included. The complete network that includes all haplotypes given in Supplementary Table 4, is shown in the bottom right panel. In both cases markers were given the following weights: DYS19 – 3, DYS385a – 2, DYS385b – 1, DYS389I – 3, DYS389II –

2, DYS390 – 2, DYS391 – 4, DYS392 – 5, DYS393 – 4, DYS439 – 2. Extended information related to the networks (population names, color codes, number of samples and Y-STRs used) can be found in Supplementary Table 4.

Supplementary Figure 5. Y-STR Median Networks for haplogroup N-Tat in Belarusian Lipka Tatars and other Eurasian populations. The network was constructed based on 10-loci haplotypes. Haplotypes differing from BLT haplotypes by no more than 1 mutation step were included. The complete network that includes all haplotypes given in Supplementary Table 4, is shown in the bottom right panel. In both cases markers were given the following weights: DYS19 – 3, DYS385a – 5, DYS385b – 2, DYS389I – 3, DYS389II – 3, DYS390 – 4, DYS391 – 3, DYS392 – 3, DYS393 – 3, DYS439 – 4. Extended information related to the networks (population names, color codes, number of samples and Y-STRs used) can be found in Supplementary Table 4.

Supplementary Figure 6. Y-STR Median Networks for haplogroup Q-M346 in Belarusian Lipka Tatars and other Eurasian populations. The network was constructed based on 10-loci haplotypes**.** Haplotypes differing from BLT haplotypes by no more than 3 mutation steps were included. The complete network that includes all haplotypes given in Supplementary Table 4, is shown in the bottom right panel. In both cases markers were given the following weights: DYS19 – 4, DYS385a – 3, DYS385b – 1, DYS389I – 3, DYS389II – 2, DYS390 – 3, DYS391 – 5, DYS392 – 4, DYS393 – 4, DYS439 – 3. Extended information related to the networks (population names, color codes, number of samples and Y-STRs used) can be found in Supplementary Table 4.

Supplementary Figure 7. Y-STR Median Networks for haplogroup R1a-M458 in Belarusian Lipka Tatars and other Eurasian populations. The network was constructed based on 14-loci haplotypes. Haplotypes differing from BLT haplotypes by no more than 1 mutation steps were included. One haplotype (indicated by a red arrow) that is the closest to

the outlier BLT haplotypes was added although it does not meet the mentioned criteria. The complete network that includes all haplotypes given in Supplementary Table 4, is shown in the bottom right panel. In both cases markers were given the following weights: DYS19 – 2, DYS389I – 4, DYS389II – 3, DYS390 – 4, DYS391 – 4, DYS392 – 5, DYS393 – 5, DYS437 – 5, DYS438 – 5, DYS439 – 2, DYS448 – 5, DYS456 – 2, DYS458 – 2, Y-GATA-H4 – 4. Extended information related to the networks (population names, color codes, number of samples and Y-STRs used) can be found in Supplementary Table 4.

Supplementary Figure 8. Y-STR Median Networks for haplogroup R1a-M558 in Belarusian Lipka Tatars and other Eurasian populations. The network was constructed based on 16-loci haplotypes. Haplotypes differing from BLT haplotypes by no more than 2 mutation steps were included. The complete network that includes all haplotypes given in Supplementary Table 4, is shown in the bottom right panel. In both cases markers were given the following weights: DYS19 – 3, DYS385a – 3, DYS385b – 2, DYS389I – 3, DYS389II – 3, DYS390 – 3, DYS391 – 3, DYS392 – 5, DYS393 – 5, DYS437 – 5, DYS438 – 5, DYS439 – 3, DYS448 – 4, DYS456 – 2, DYS458 – 2, Y-GATA-H4 – 3. Extended information related to the networks (population names, color codes, number of samples and Y-STRs used) can be found in Supplementary Table 4.

Supplementary Figure 9. Y-STR Median Networks for haplogroup R1a-Z2125 in Belarusian Lipka Tatars and other Eurasian populations. The network was constructed based on 17-loci haplotypes. Haplotypes differing from BLT haplotypes by no more than 2 mutation steps were included. Three haplotypes (indicated by red arrows) closest to the outlier BLT haplotypes were added although it does not meet the mentioned criteria. The complete network that includes all haplotypes given in Supplementary Table 4, is shown in the bottom right panel. In both cases markers were given the following weights: DYS19 – 3, DYS385a – 5, DYS385b – 3, DYS389I – 3, DYS389II – 3, DYS390 – 3, DYS391 – 3, DYS392 – 5,

DYS393 – 5, DYS437 – 5, DYS438 – 5, DYS439 – 4, DYS448 – 3, DYS456 – 3, DYS458 – 3, DYS635 – 4, Y-GATA-H4 – 3. Extended information related to the networks (population names, color codes, number of samples and Y-STRs used) can be found in Supplementary Table 4.

Supplementary Figure 10. Y-STR Median Networks for haplogroup R1b-M478 (=M73) in Belarusian Lipka Tatars and other Eurasian populations. The network was constructed based on 8-loci haplotypes. All haplotypes given in Supplementary Table 4 are included. Markers were given the following weights: DYS19 – 5, DYS389I – 3, DYS389II – 2, DYS390 – 1, DYS391 – 4, DYS392 – 5, DYS393 – 4, DYS439 – 3. Extended information related to the networks (population names, color codes, number of samples and Y-STRs used) can be found in Supplementary Table 4.

Supplementary Figure 11. Phylogenetic trees of mtDNA haplogroups C4a; D4j, D4g, D2; G2a including new complete sequences generated in this study. (Excel spreadsheet)

Supplementary Figure S12. FineSTRUCTURE tree of genetic groups. The tree is constructed by successive merging of pairs of clusters keeping the highest value of posterior probability among all possible pairs to merge. Numbers along branches of the tree indicate posterior probability.

Supplementary Figure 13. ChromoPainter chunkcounts coancestry matrix. Each row ("recipient") represents a composition of haplotypes (total counts) received from "donor" individuals in columns. Lower triangle – population coancestry matrix – averaged values of total chunkcounts for individuals assigned to a particular genetic cluster; upper triangle – individual coancestry matrix. Rows and columns are arranged according to fineSTRUCTURE clustering analysis (Supplementary Figs. 14, 12).

Supplementary Figure 14. FineSTRUCTURE pairwise coincidence matrix. Plots, obtained from two independent runs (bottom right – run 1; top left – run 2), are characterized by high symmetry. Colors indicate the posterior coincidence probability.

Supplementary Figure 15. Example of coancestry curves used in the GLOBETROTTER analysis to infer the sources and dates of admixture in Belarusian Lipka Tatars. (A) Null.ind1. (B) Null.ind0. Plots in the left column show relative probability of jointly copying two chunks from donor clusters Slavs73 and MongXibo14 at different genetic distances, in Belarusian Lipka Tatars. The negative slope of the curve suggests that Slavs73 and MongXibo14 donor clusters contribute to different sites of the admixture. Coancestry curves in the middle and right columns show examples of donor clusters that contribute to the same site of the admixture in Belarusian Lipka Tatars.

Supplementary Figure 16. Runs of homozygosity (RoH) in Belarusian Lipka Tatars. (A) Plot shows natural logarithm of mean sum of RoH lengths (x-axis) plotted *vs* mean number of RoH (y-axis). Values for six Belarusian Lipka Tatars (Blt1-Blt6) and the population mean (Blt_MEAN) are plotted separately. **(B)** Plot shows the share of individuals in each population sample, having natural logarithm values of mean sum of RoH length as high as or higher than the mean value for Belarusian Lipka Tatars (red bar).

Supplementary Figure 17. Map of Belarus showing origin of samples of Belarusian Lipka Tatars in this study. 1 – Grodna, 2 – Shchuchyn, 3 – Iuje, 4 – Ashmyany, 5 – Kreva, 6 – Smarhon, 7 – Maladzyechna, 8 – Pastavy, 9 – Hlybokae, 10 – Dokshytsy, 11 – Minsk, 12 – Smilavichy, 13 – Uzda. The original map of Belarus (https://be-tarask.wikipedia.org/wiki/%D0%A4%D0%B0%D0%B9%D0%BB:Map_Of_Belarus_blank_2.svg) is licensed under the Attribution-Share-Alike 3.0 Unported license. The license terms can be found on the following link: https://creativecommons.org/licenses/by-sa/3.0/.

Supplementary Figure 18. (A) ADMIXTURE plot (k2-k10). (B) Box and whiskers plot of the cross validation (CV) indexes of all runs of the ADMIXTURE analysis. (C) Variation in log-likelihood (LL) scores in 5% (dark green), 10% (middle green), 20% (light green) fractions of runs that reached the highest LLs. We assume that a global LL maximum was achieved at a given k if 10% of the runs with the highest LL score showed minimal variation in LL scores (i.e. less than 1 LL unit). Thus, the global LL maximum was reached at k 2-10. The lowest cross-validation error (indicates the best predicted k in the ADMIXTURE model) was for k 6.

Supplementary Information Tables Legends

Supplementary Table 1. Population abbreviations.

Supplementary Table 2. Confidence Intervals (CI) for Y-chromosome haplogroup frequencies in BLT and Belarusians.

Supplementary Table 3. Y-STR haplotypes generated in this study.

Supplementary Table 4. Y-STR Median Networks extended information.

Supplementary Table 5. Matrix of population pairwise R_{ST} for hg Q1a-M346.

Supplementary Table 6. Matrix of population pairwise R_{ST} for hg R1a-Z2125.

Supplementary Table 7. Matrix of population pairwise R_{ST} for hg G2a-U1.

Supplementary Table 8. Matrix of population pairwise R_{ST} for hg J1-P58.

Supplementary Table 9. Matrix of population pairwise R_{ST} for hg J2a(xM67).

Supplementary Table 10. Matrix of population pairwise R_{ST} for hg R1a-M458.

Supplementary Table 11. Matrix of population pairwise R_{ST} for hg R1a-M558.

Supplementary Table 12. MtDNA HVSI haplotypes in Belarusian Lipka Tatars.

Supplementary Table 13. Matrix of the average population pairwise F_{ST} based on whole genome SNP data variation.

Supplementary Table 14. Populations used in whole genome SNP analyses.

Supplementary Table 15. List of population pairs with negative f3-statistics for Belarusian Lipka Tatars.

Supplementary Table 16. Results of the GLOBETROTTER analysis.

Supplementary Table 17. fineSTRUCTURE genetic clusters.

Supplementary Table 18. Admixture sources and dates inferred by the ALDER algorithm.

Supplementary Table 19. Belarusian Lipka Tatar tribal names shared by Turkic and Mongolic peoples.

Supplementary Table 20. DNA samples used in this study.

Supplementary Table 21. Y-chromosome biallelic markers genotyped in this study.

Supplementary Table 22. Complete mtDNA haplotypes generated in this study.

Supplementary Fig. 1

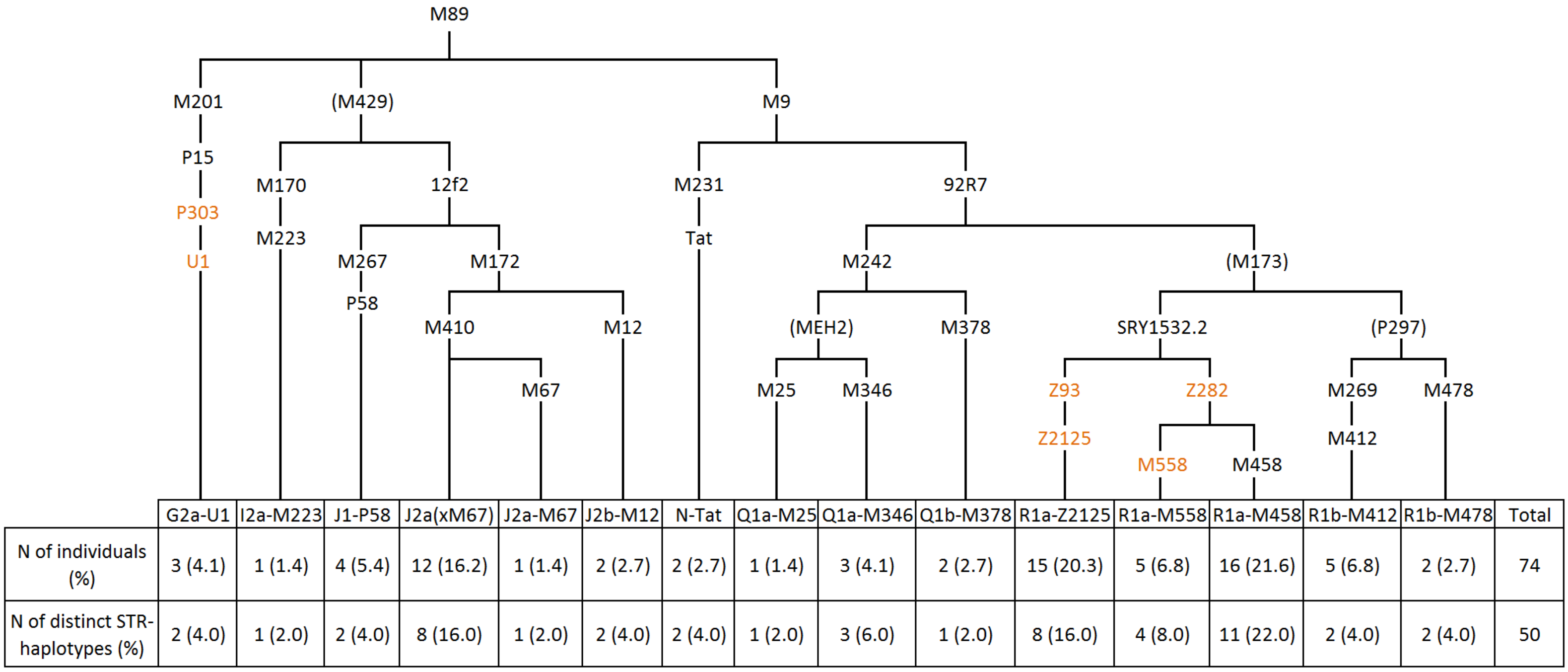

	G2a-U1	I2a-M223	J1-P58	J2a(xM67)	J2a-M67	J2b-M12	N-Tat	Q1a-M25	Q1a-M346	Q1b-M378	R1a-Z2125	R1a-M558	R1a-M458	R1b-M412	R1b-M478	Total
N of individuals (%)	3 (4.1)	1 (1.4)	4 (5.4)	12 (16.2)	1 (1.4)	2 (2.7)	2 (2.7)	1 (1.4)	3 (4.1)	2 (2.7)	15 (20.3)	5 (6.8)	16 (21.6)	5 (6.8)	2 (2.7)	74
N of distinct STR-haplotypes (%)	2 (4.0)	1 (2.0)	2 (4.0)	8 (16.0)	1 (2.0)	2 (4.0)	2 (4.0)	1 (2.0)	3 (6.0)	1 (2.0)	8 (16.0)	4 (8.0)	11 (22.0)	2 (4.0)	2 (4.0)	50

Supplementary Fig. 2

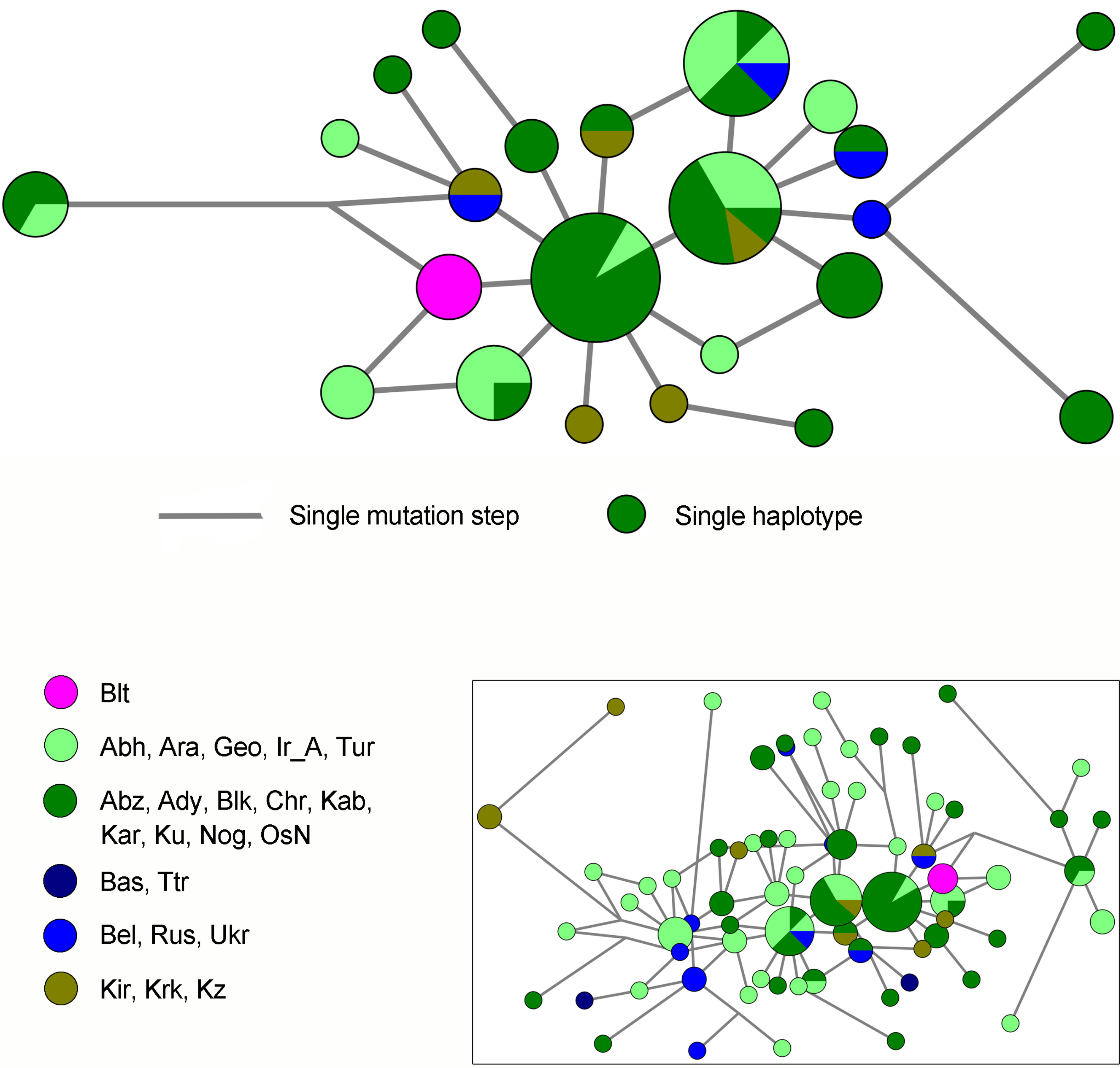

Supplementary Fig. 3

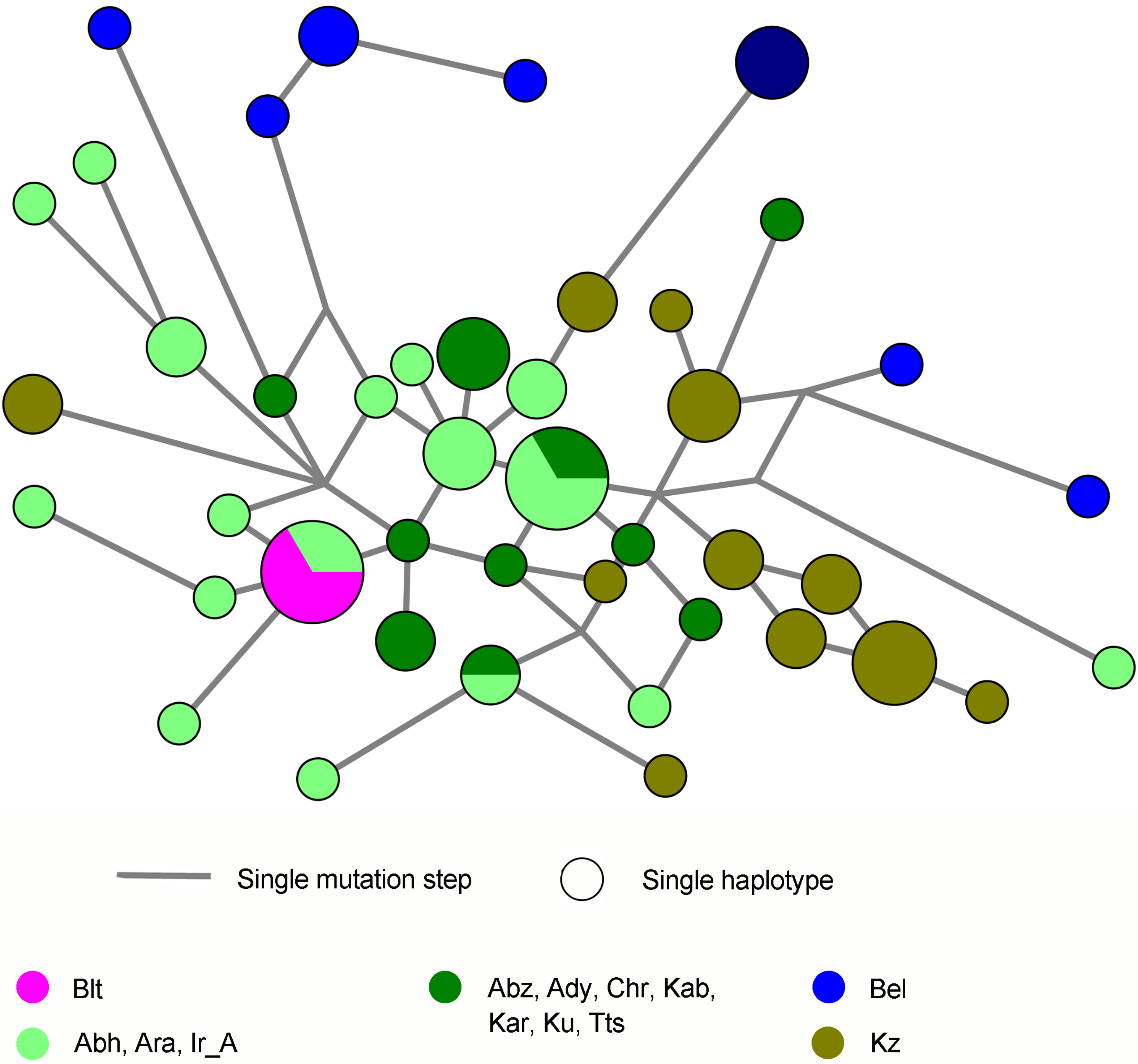

Supplementary Fig. 4

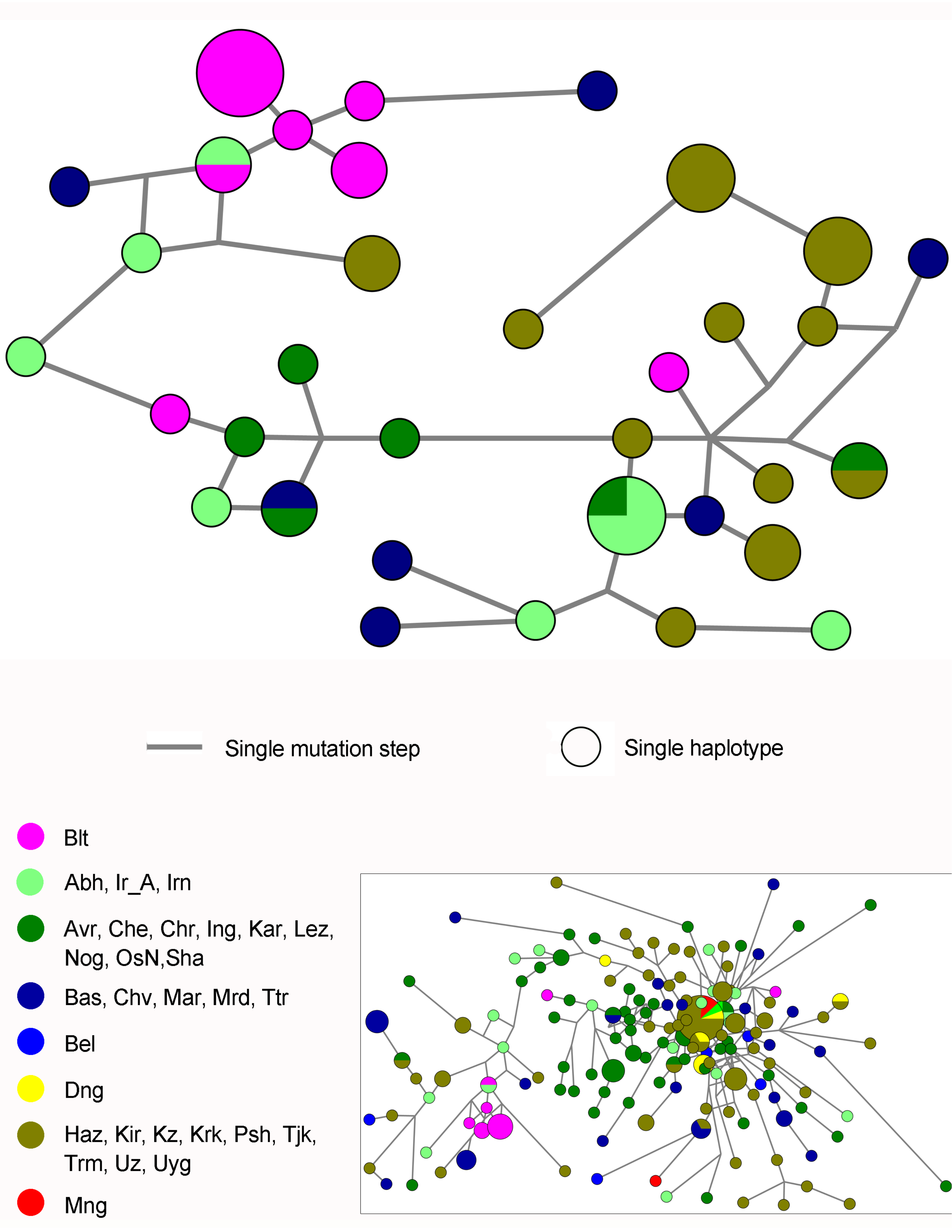

Supplementary Fig. 5

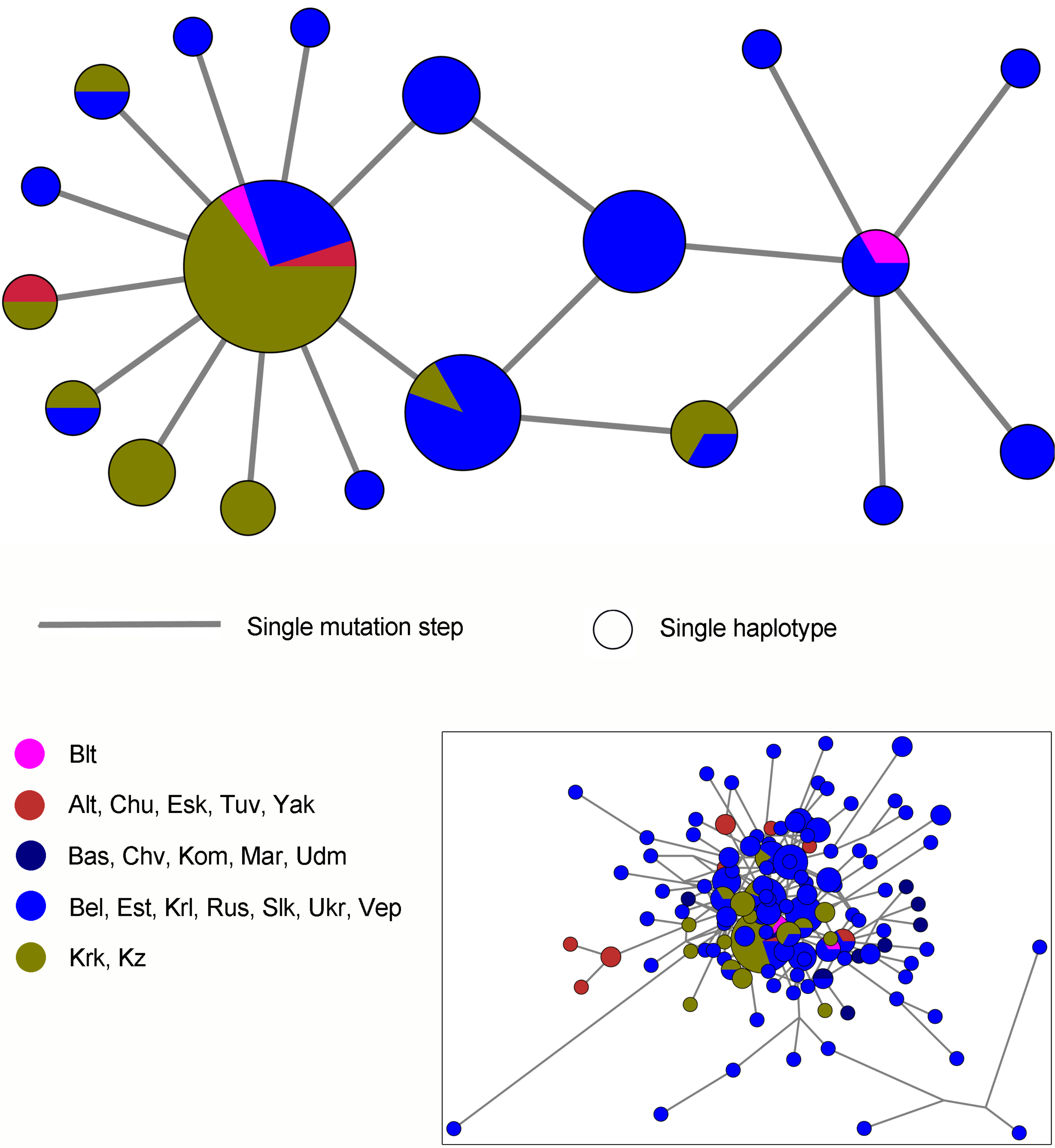

Supplementary Fig. 6

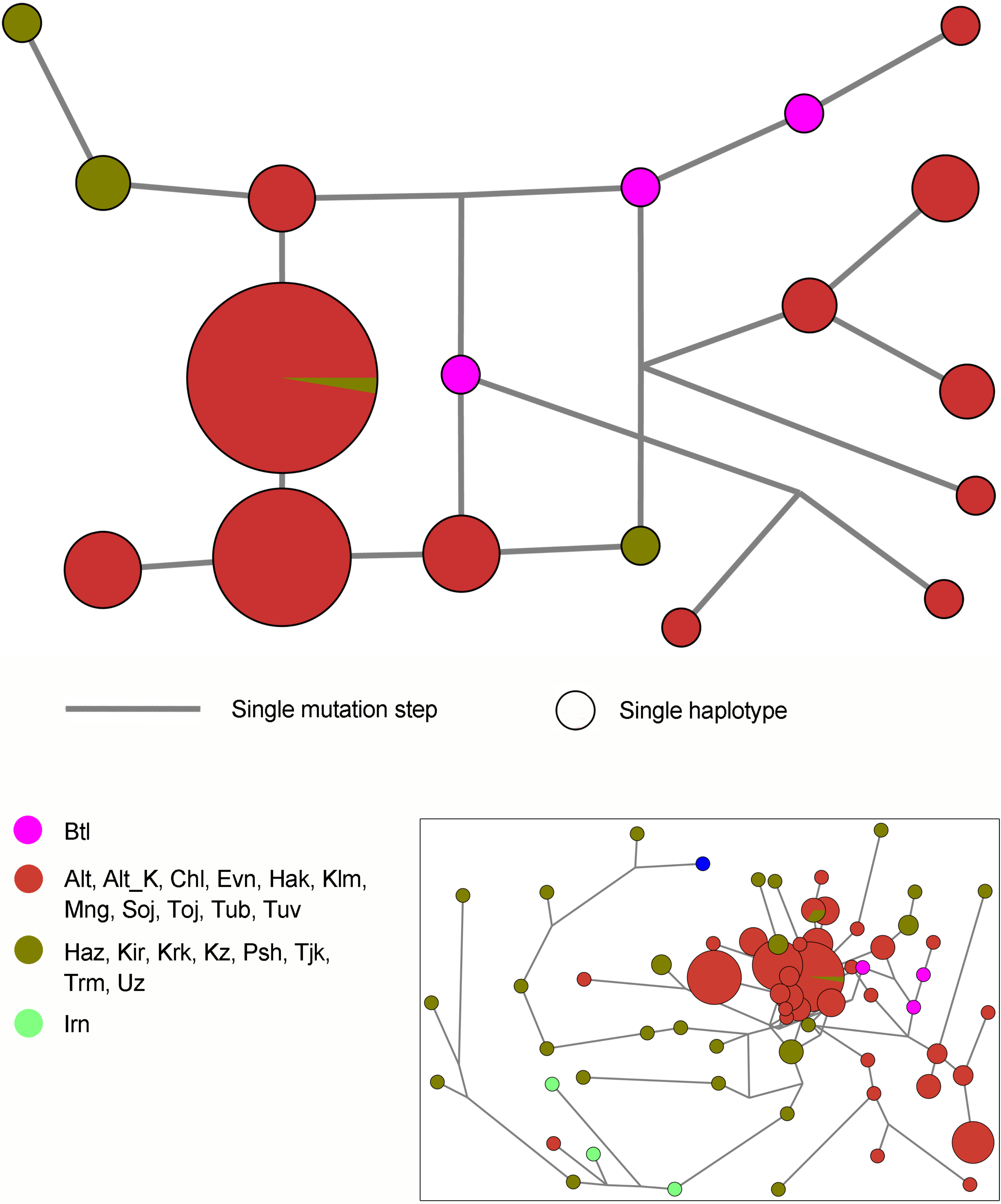

Supplementary Fig. 7

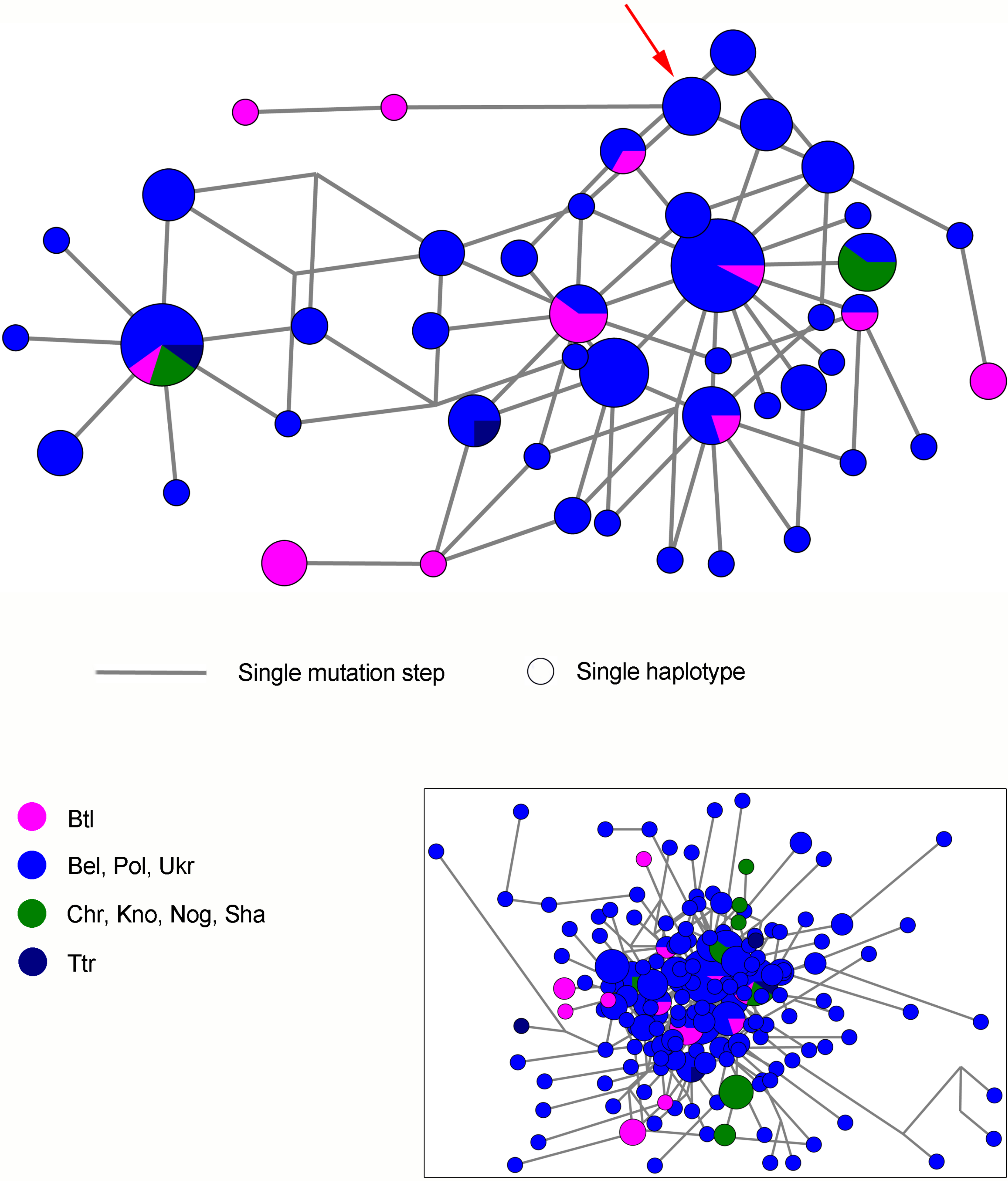

Supplementary Fig. 8

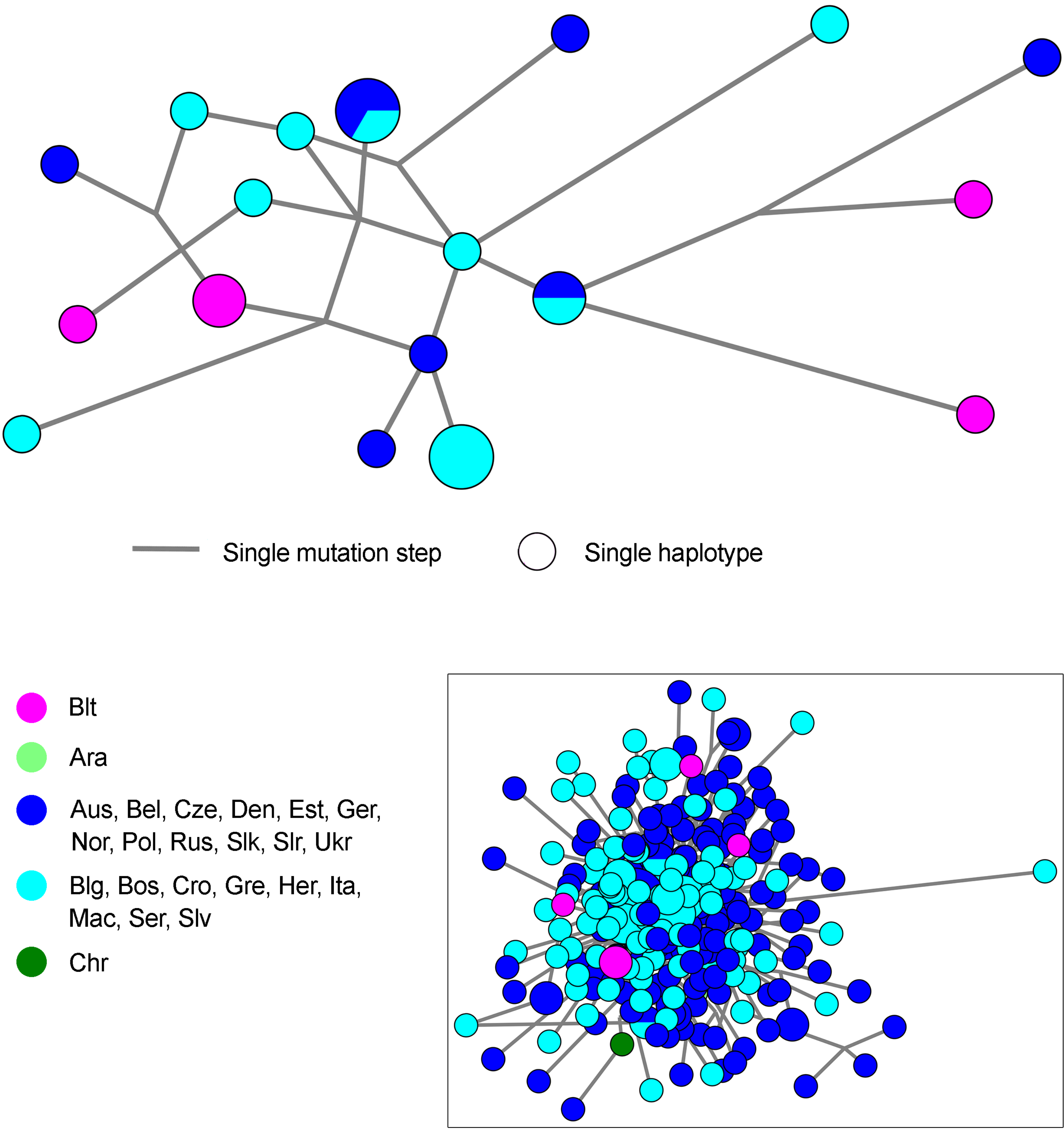

Supplementary Fig. 9

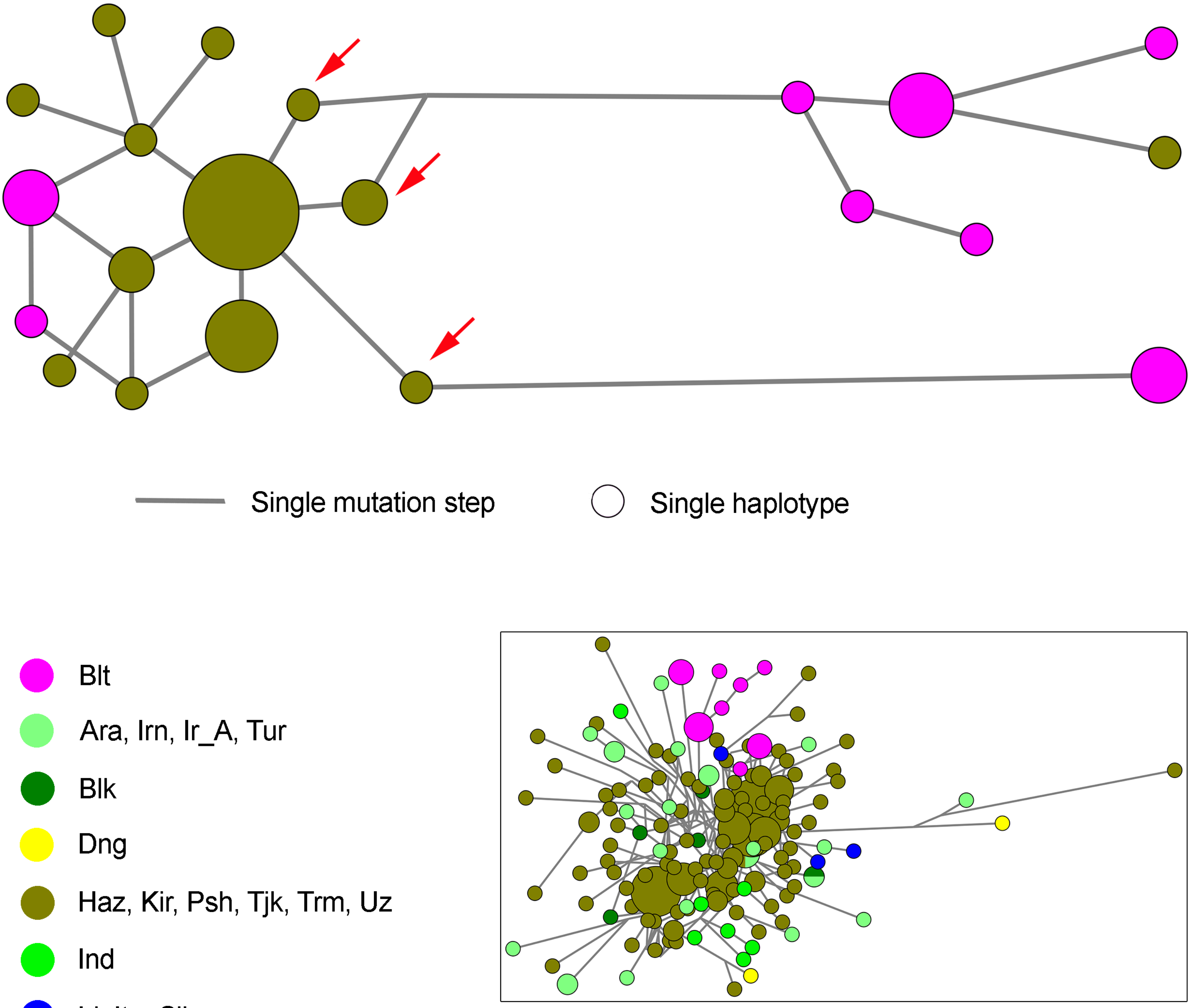

Supplementary Fig. 10

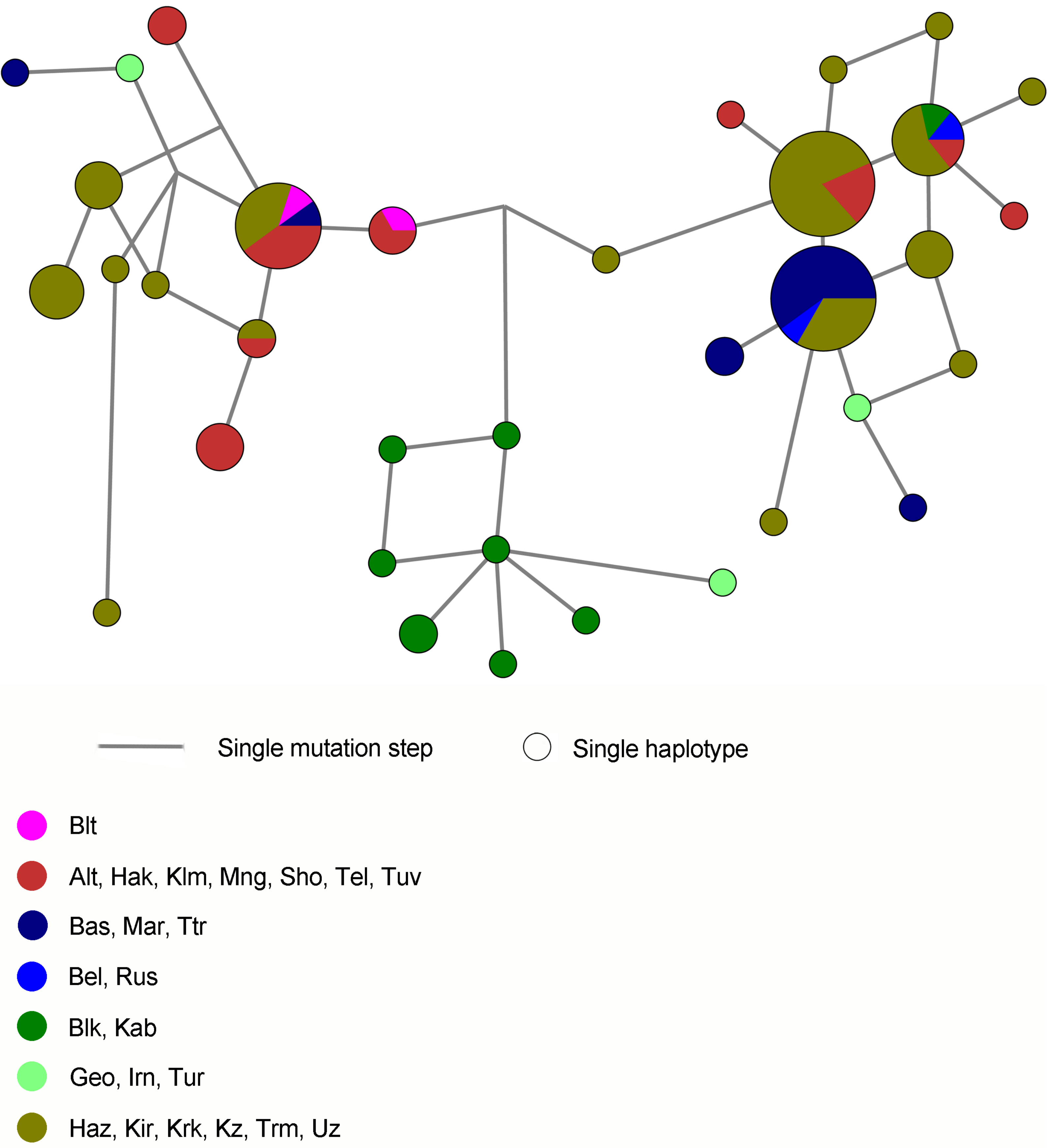

Supplementary Fig. 12

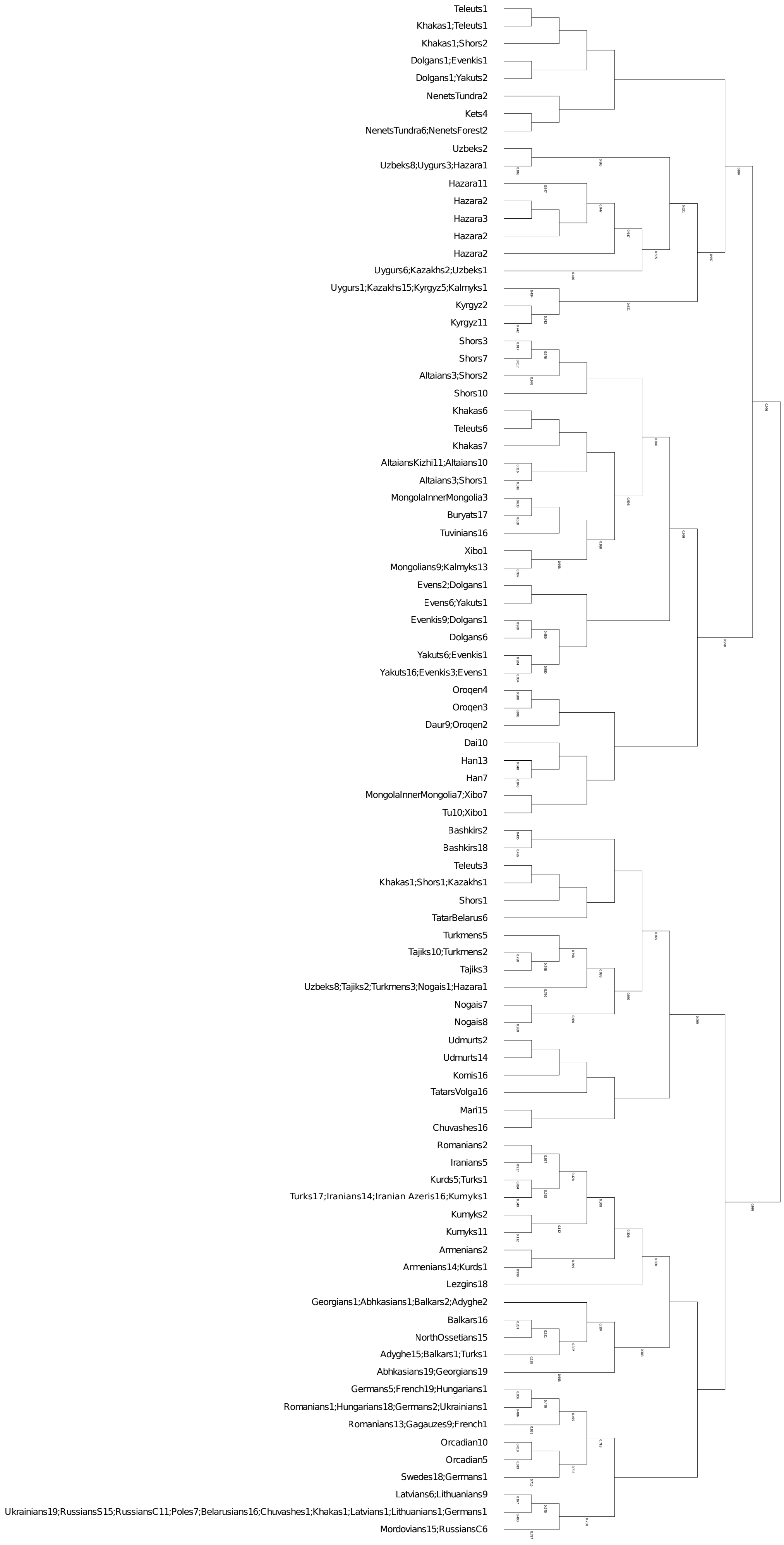

Supplementary Fig. 13

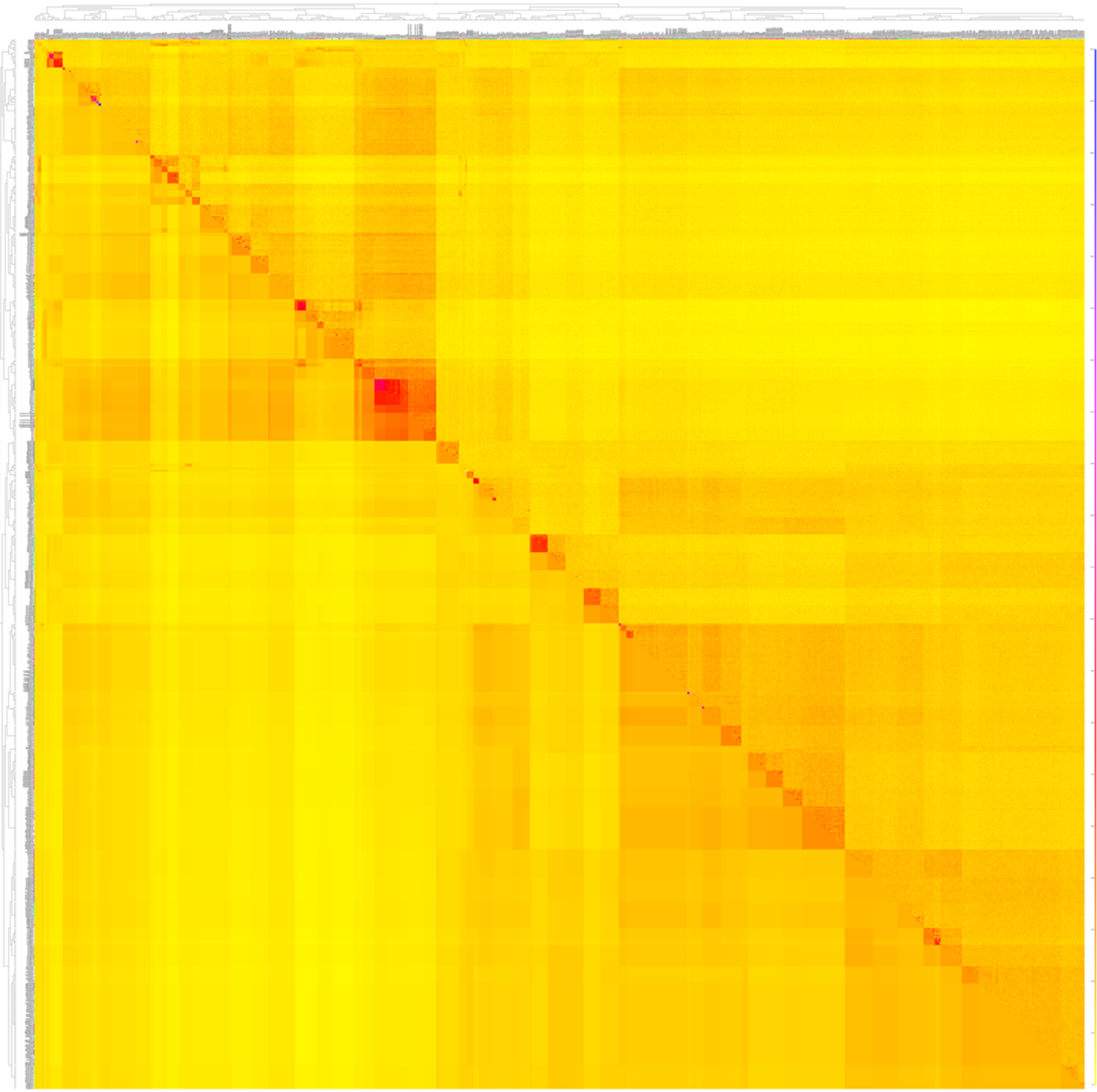

Supplementary Fig. 14

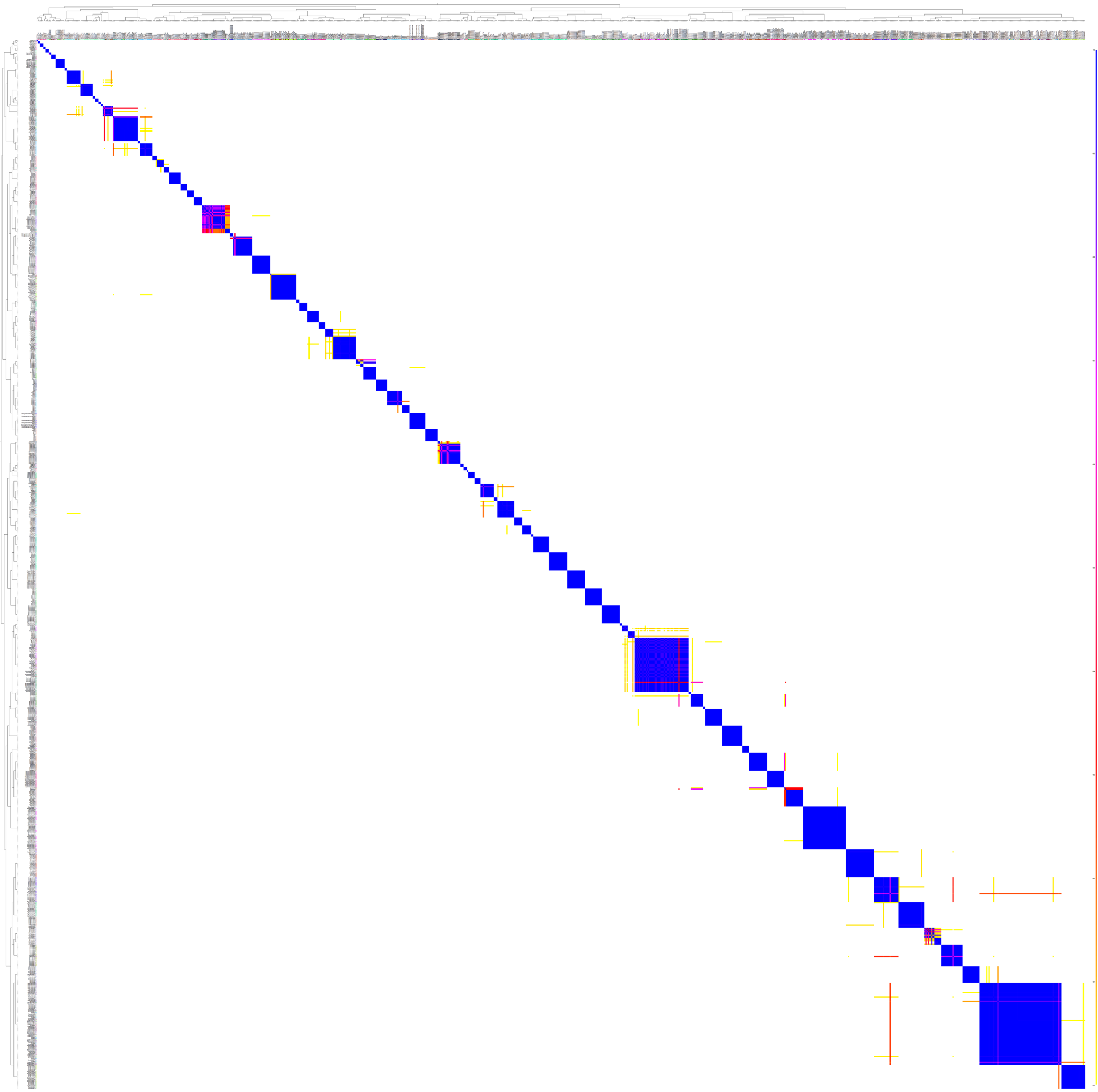

Supplementary Fig. 15

A

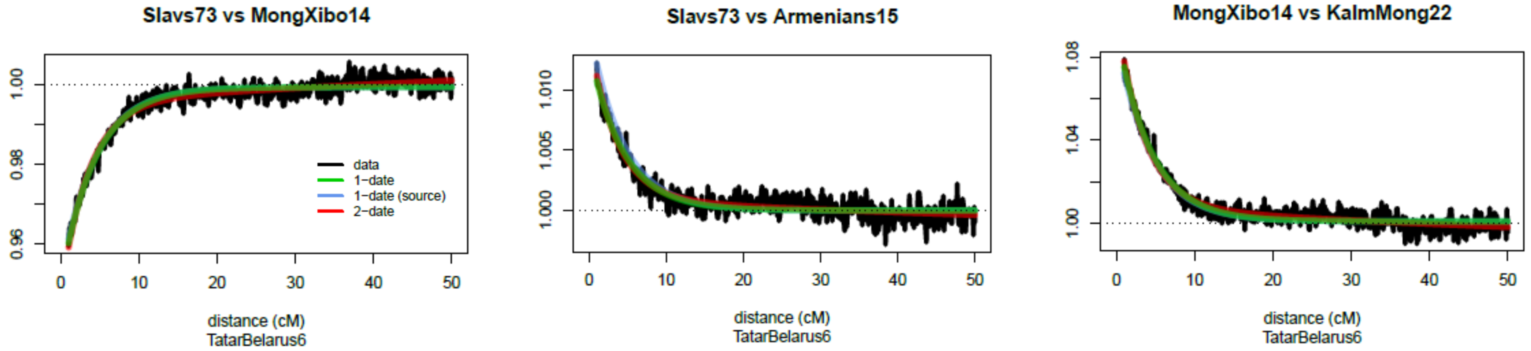

B

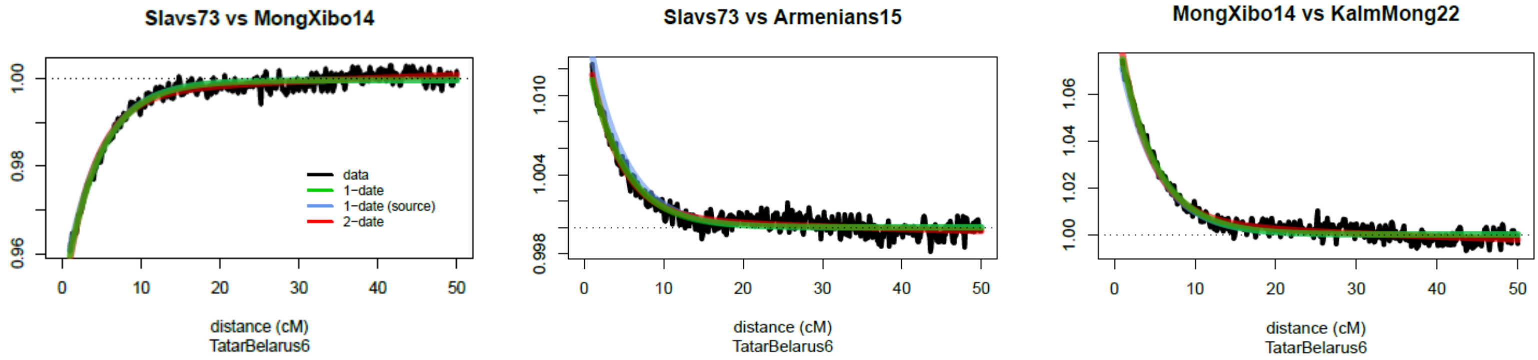

Supplementary Fig. 16

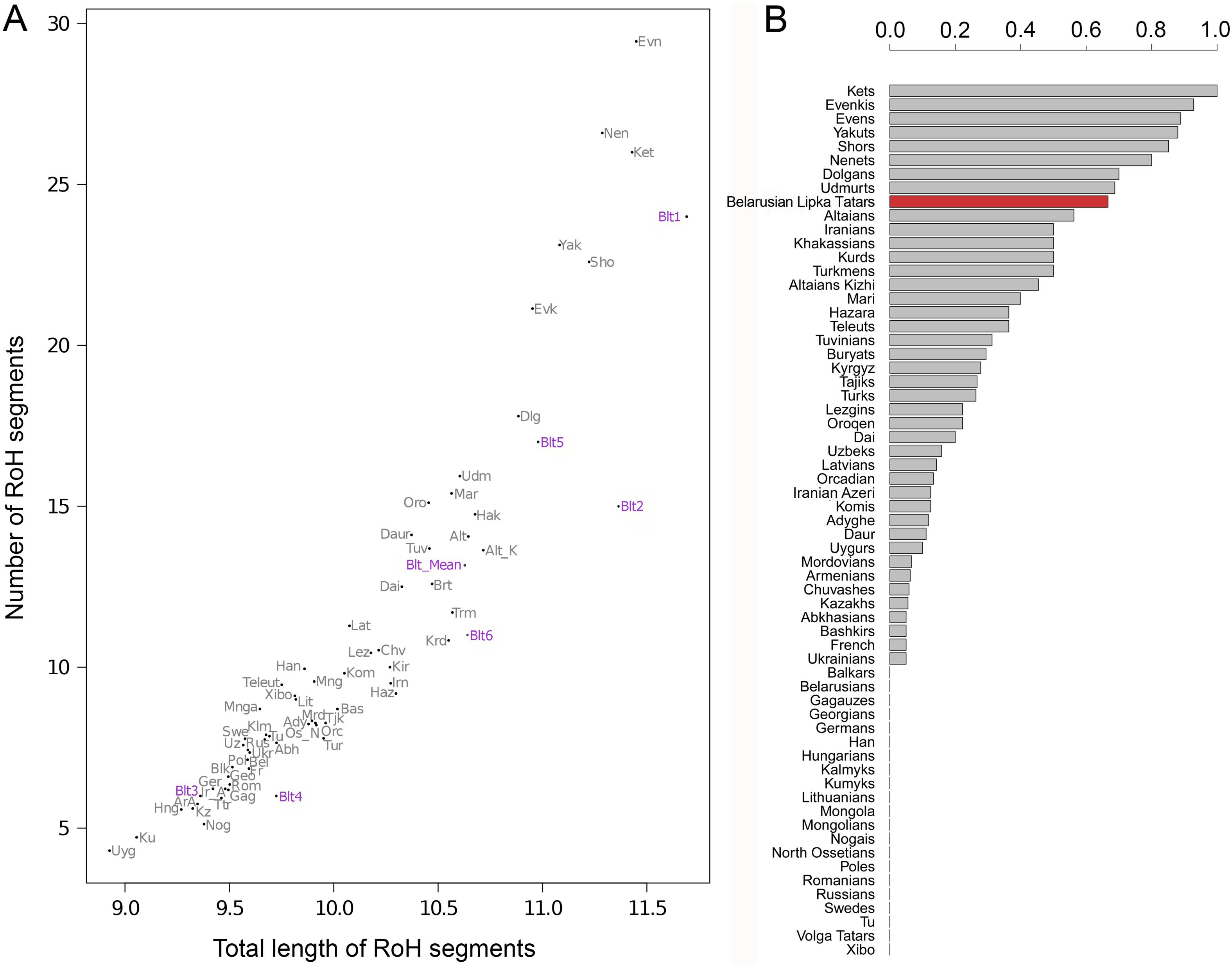

Supplementary Fig. 17

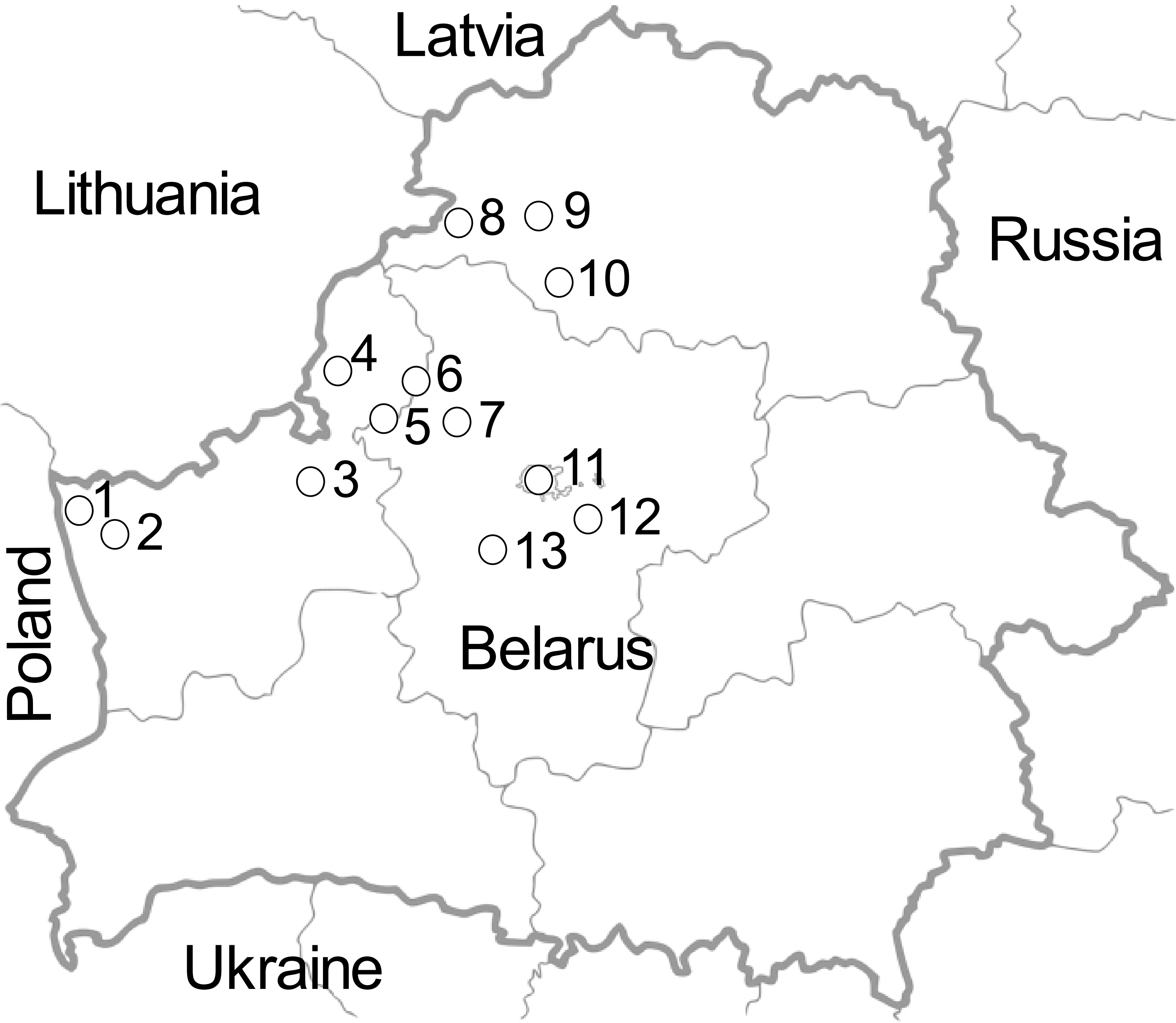

Supplementary Fig. 18

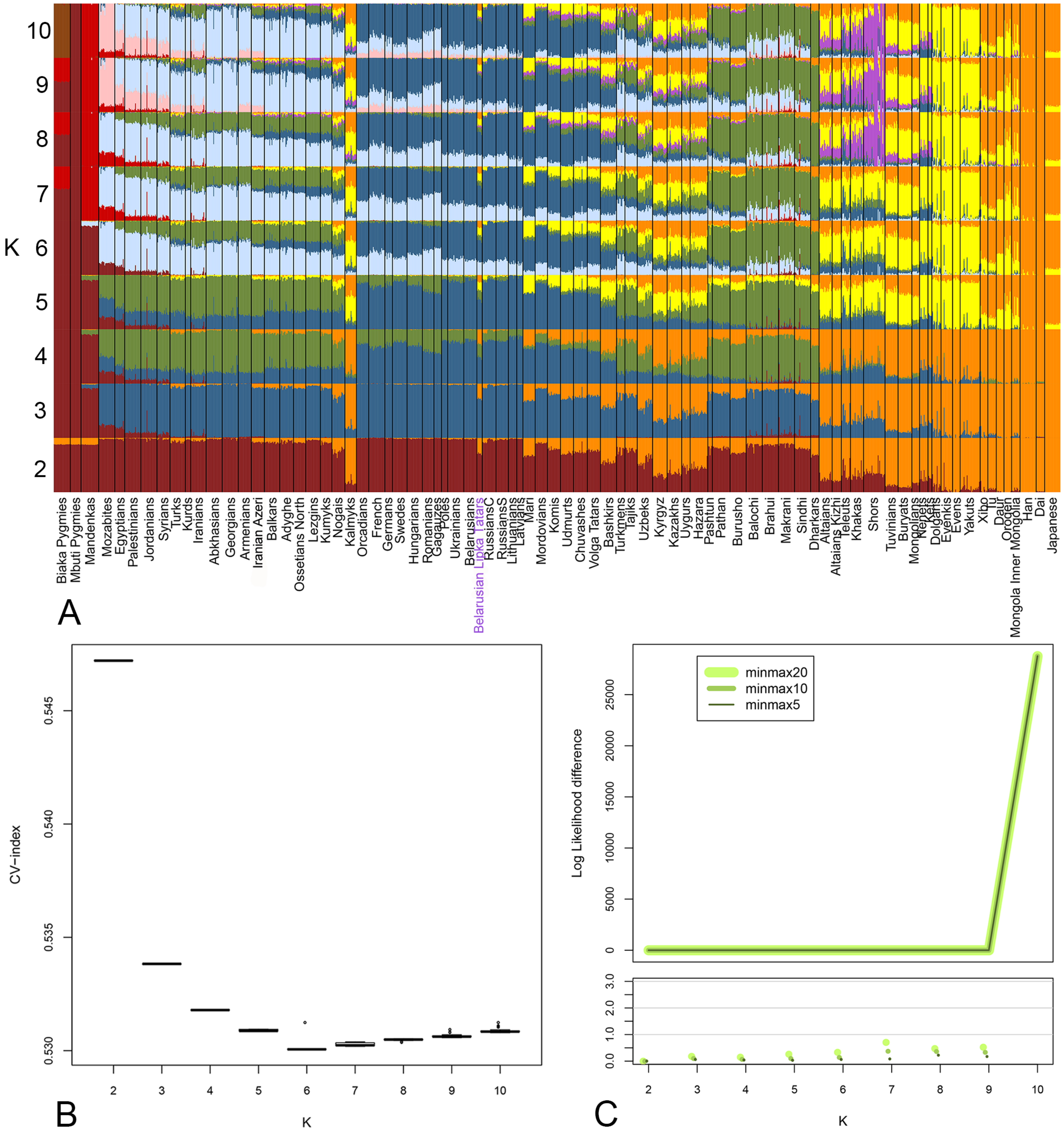

Supplement: Supplementary Information [file srep30197-s1.pdf]
